# When Does Score Fusion Help? Conformally Certified Out-of-Distribution Detection for Camera and LiDAR Sensors

**DOI:** 10.3390/s26154706

**Published:** 2026-07-24

**Authors:** Loránt Szabó, Zoltán Weltsch, Andrea Ádámné-Major

**Affiliations:** 1 AI Research Center, John von Neumann University, 6000 Kecskemét, Hungary; major.andrea@nje.hu; 2 Department of Road and Rail Vehicles, Széchenyi István University, 9026 Győr, Hungary; weltsch.zoltan@sze.hu

**Keywords:** camera-sensor reliability, LiDAR sensor-anomaly detection, out-of-distribution detection, Fisher’s method, conformal prediction, PAC bounds, sensor data integrity, safety-critical perception, statistical score fusion

## Abstract

**Highlights:**

**What are the main findings?**

- A single Fisher-fusion + ECDF-calibration + PAC–Hoeffding-bound pipeline applies uniformly to camera (PUG) and LiDAR (nuScenes) sensors, providing distribution-free finite-sample FPR95 control suitable for ISO 26262 safety-case construction.
- Uniform *p*-value score fusion gives at most a small gain over the best single detector (camera: the dominant Mahalanobis is diluted; LiDAR: a small +0.012, calibration-sensitive gain). The one substantial, robust fusion gain is cross-backbone variance-standardised *z*-averaging on the camera (+0.0233), which exploits structurally independent backbones.

**What are the implications of the main findings?**

- Sensor-data-integrity monitoring can be deployed with a single, reusable, PAC-certifiable scoring pipeline across heterogeneous sensor modalities.
- The empirical AUROC value of score fusion is limited and regime-specific: uniform *p*-value combination is dominated by the best single detector across both sensors, so deployments should not assume a fusion gain. The reusable contribution is the distribution-free FPR certificate, which holds for any fused (or single) statistic and transfers across modalities.

**Abstract:**

Camera and LiDAR sensors in safety-critical autonomous systems suffer undetected distributional shifts that silently corrupt downstream perception. Out-of-distribution (OOD) detection is the established sensor-data-integrity primitive, but no single post hoc detector covers every shift type, and existing detectors lack guarantees on their false-positive rate (FPR). This paper asks *when* calibrated score fusion helps and provides a distribution-free finite-sample FPR certificate. Four post hoc scores—Maximum Softmax Probability (MSP), Energy, Mahalanobis distance and *k*-nearest-neighbour (KNN) distance—are calibrated to *p*-values by the empirical cumulative distribution function (ECDF) and combined by Fisher’s method or cross-backbone *z*-score averaging, then wrapped in a conformal predictor with Hoeffding-based Probably Approximately Correct (PAC) bounds. On the full-split PUG camera benchmark (215,040 images), uniform same-backbone *p*-value fusion does *not* beat the best single detector (Mahalanobis); the gain comes from cross-backbone diversity: a *z*-score average of Mahalanobis distances over ResNet-50 and frozen DINOv2 reaches a mean area-under-the-ROC-curve (AUROC) of 0.9258 (+0.0199), rising to 0.9292 (+0.0233) with added spectral and dropout signals (DeLong $p<10^{-9}$). On the nuScenes LiDAR sensor (256,873 frames), uniform fusion yields only a small, calibration-sensitive gain over the best single detector (MSP), so the substantial fusion gain is confined to cross-backbone averaging on the camera. The distribution-free PAC certificate, by contrast, transfers across both sensors with margins below 1.5% (0.96% camera, 0.25% LiDAR), giving evidence usable in ISO 26262 and EASA CoDANN safety cases.

## 1. Introduction

### 1.1. Sensor Data Integrity in Safety-Critical Perception

Autonomous-driving perception stacks ingest data from heterogeneous sensors—cameras for semantic perception, LiDAR for geometric reasoning, radar for adverse-weather robustness—and feed it through deep neural classifiers and detectors that are trained under a closed-world assumption [1]. In real-world deployment this assumption is systematically violated: sensors encounter operating conditions outside their calibration envelope (rainy weather, low illumination, sensor fouling, novel obstacles), and the downstream classifier silently produces overconfident predictions on inputs it was never trained to handle [2]. Detecting such inputs at the sensor-data-integrity layer—i.e., flagging frames whose statistical signature deviates from the calibrated in-distribution sensor stream—is the established remedy in safety-critical perception [1,3].

The difficulty is compounded by the heterogeneity of distributional shift. *Covariate shift* alters the appearance of sensor inputs while preserving their semantic category (e.g., weather changes in driving scenes, sensor fouling, low-light conditions). *Semantic shift* introduces entirely new categories absent from the calibration set (e.g., an animal on a highway, an unseen vehicle class in a LiDAR point cloud). *Combined out-of-distribution* (OOD) conditions present both shift types simultaneously. These distinct failure modes demand different detection mechanisms, yet no single method reliably handles all shift types [1,3].

### 1.2. The Fundamental Single-Detector Limitation

Post hoc sensor-anomaly scoring functions, applied to a pretrained perception module without retraining, span several families. Confidence-based scores such as Maximum Softmax Probability (MSP) [2] and Energy Score [4] leverage output-layer statistics of the deployed classifier but suffer from overconfidence under subtle covariate changes in the sensor stream. Distance-based scores such as the Mahalanobis distance [5] measure proximity to in-distribution clusters in feature space, but can struggle with semantically novel inputs. Non-parametric approaches such as *k*-nearest neighbours (KNN) [6] avoid distributional assumptions but require careful feature normalisation, which is particularly relevant for sparse LiDAR-feature spaces. Vision-language detectors based on Contrastive Language–Image Pre-training (CLIP) [7] excel at semantic novelty on camera input, but exhibit systematic blindness to covariate shifts [8] and do not generalise to non-image sensor modalities.

**Figure 1:**
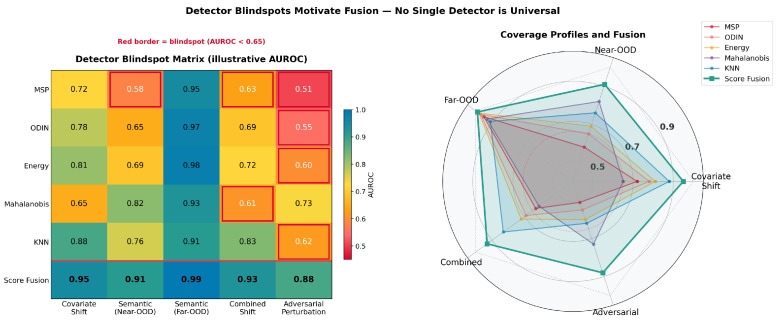
Motivation for statistical fusion (illustrative AUROC values): different OOD detection methods exhibit complementary failure modes (blindspots) under covariate and semantic shifts, so relying on any single detector leaves the system vulnerable. The quantitative analysis of when fusion actually closes these blindspots is given in Section 4, Section 5, Section 6 and Section 7.

As visualised in Figure 1, this complementarity of failure patterns is not merely an empirical nuisance; it represents a fundamental limitation. Comprehensive evaluations confirm that “no single score is useful for detecting different kinds of OOD instances” [1], which motivates the principled fusion of heterogeneous sensor-anomaly scores rather than reliance on any individual detector. Furthermore, although the experiments using the parametrisable PUG benchmark naturally emphasise synthetic covariates to allow strict isolation of shift topologies on the camera modality, the underlying findings robustly translate both to real-world autonomous driving safety requirements and, as demonstrated in Section 7, to LiDAR-sensor data integrity on the nuScenes benchmark.

### 1.3. Statistical Fusion as a Principled Solution

This study investigates score-level fusion that transforms heterogeneous OOD scores into a unified, calibrated detection statistic, and it asks under what conditions such fusion improves on the best single detector. Although raw scores such as MSP, Energy, and Mahalanobis distance originate from the same backbone architecture, they capture structurally distinct representational spaces (logits versus intermediate geometric features), providing approximately complementary, though not strictly independent, signals. The framework operates in three stages: (1) each raw detector score is mapped to a *p*-value via the Empirical Cumulative Distribution Function (ECDF) fitted on in-distribution calibration data, resolving the scale-incompatibility problem [9]; (2) the resulting *p*-values are combined by an aggregator—we study Fisher’s method [10], the closely related Stouffer *Z*-method [11], and a cross-backbone *z*-score average—and our central empirical finding is that this choice is decisive (Section 4); and (3) the fused statistic is augmented with conformal prediction and PAC–Hoeffding bounds to provide provable, distribution-free FPR control that holds regardless of the aggregator [12,13]. The empirical-null calibration of Section 2 removes the need to assume mutual independence of the fused *p*-values.

### 1.4. Contributions

**Figure 2:**
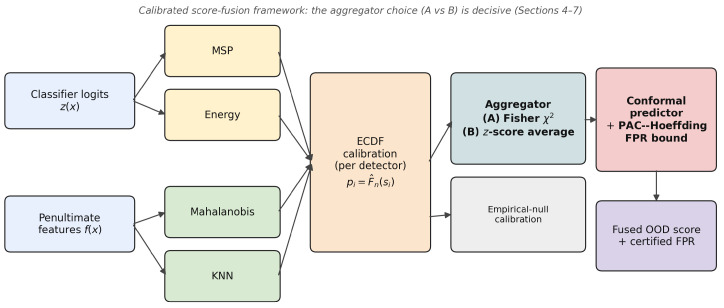
The ECDF-calibrated score-fusion framework studied in this paper. Raw classifier logits and penultimate-layer features feed parallel post hoc detector pipelines (MSP, Energy, Mahalanobis, KNN), each calibrated to a *p*-value via the in-distribution ECDF. The calibrated scores are then combined by one of two aggregators—Fisher’s chi-squared statistic or a cross-backbone *z*-score average—and the fused statistic is wrapped in a conformal predictor with a Hoeffding-based PAC bound on the FPR. A central finding (Section 4, Section 5, Section 6 and Section 7) is that the aggregator choice is decisive: uniform *p*-value combination yields at most a small gain over the best single detector, whereas cross-backbone *z*-score averaging yields the substantial, robust gain (camera, +0.0233). Colour coding: blue: inputs; yellow: logit-based detectors; green: feature-based detectors; orange: ECDF calibration; teal: aggregators; grey: empirical-null calibration; red: conformal–PAC certification; purple: certified output.

The unified architecture of the framework is presented in Figure 2.

The contributions of this paper are fivefold.

- **C1 (A Boundary Condition for Calibrated Score Fusion):** Through a full-split empirical audit on the PUG photorealistic camera-sensor benchmark ($n_{\mathrm{ID},\mathrm{val}}\;=\;1800$; $n_{\mathrm{ID},\mathrm{test}}\;=\;16,200$; $n_{\mathrm{CS}}\;=\;135,600$; $n_{\mathrm{SS}}\;=\;7200$; $n_{\mathrm{COOD}}\;=\;54,240$, totalling 215,040 images), we establish when ECDF-calibrated *p*-value fusion of post hoc sensor-anomaly scores helps and when it does not. Within-backbone fusion of every one of the eleven mixes drawn from {MSP, Energy, Mahalanobis, KNN} *dilutes* the strongest single detector (Mahalanobis); we show this is intrinsic to equal-weight *p*-value combination over heterogeneous-quality constituents (it is reproduced by both Fisher’s and Stouffer’s combiners) rather than a tuning artefact. The genuine camera gain comes instead from cross-backbone diversity: a *z*-score average of class-conditional Mahalanobis distances from the fine-tuned ResNet-50 and a frozen DINOv2-ViT-B/14 (Section 4.7) reaches mean AUROC 0.9258, +0.0199 over Mahalanobis-only, with DeLong $p<10^{-9}$ on CS and COOD and bootstrap std <0.002 on each shift.
- **C2 (Cross-Domain Validation):** The identical pipeline is applied to the Canadian Institute For Advanced Research 10-class (CIFAR-10) dataset with standard OOD benchmarks, ranking third among twelve post hoc methods on Street View House Numbers (SVHN) with AUROC = 0.976 while consistently outperforming all three constituent detectors.
- **C3 (PAC-Certified Conformal Detection—the modality-independent contribution):** A conformal prediction wrapper with Hoeffding-based PAC bounds provides provably valid, distribution-free FPR control at the 95% true-positive rate operating point (FPR95). The certificate applies to *any* fused statistic and transfers unchanged from the camera to the LiDAR sensor; with the sample count set correctly to the in-distribution (negative) test size, the empirical-to-bound margins are 0.96% on the PUG camera and 0.25% on the nuScenes LiDAR (both below 1.5%).
- **C4 (Layer-wise Detection Dynamics):** Systematic analysis across ResNet-50 stages reveals that early layers are superior for covariate-shift detection (Layer 1 AUROC = 0.813 versus Layer 4 AUROC = 0.757), whereas deeper layers favour semantic-shift detection (Layer 4 AUROC = 0.572). Naïve multi-layer fusion exhibits a documented dilution effect.
- **C5 (Cross-Modality LiDAR Validation):** The same pipeline is transferred to the nuScenes LiDAR sensor (241,346 in-distribution and 15,527 out-of-distribution point-cloud frames). The best single detector is MSP (0.8158); uniform fusion gives only a small, calibration-sensitive gain (Fisher(MSP+KNN) 0.8275, +0.012, DeLong-significant but null under held-out calibration), confirming on a second, physically distinct sensor that uniform score fusion offers at most a marginal advantage. What transfers without modality-specific re-tuning is the distribution-free certificate (C3), which holds unchanged on LiDAR; we additionally report a sensor-degradation OOD type on which a feature-space detector, not fusion, carries the signal.

### 1.5. Method Landscape Positioning

Table 1 positions the proposed approach within the broader OOD detection landscape, summarising approximately seventy methods across eight categories from the OpenOOD v1.5 benchmark [1,3]. The “Fused” column marks methods that combine heterogeneous detector families at the score level (a property distinct from ensembling, which averages same-family models); “Cert.” marks methods that issue a finite-sample bound on their empirical FPR.

## 2. Background and Methodology

### 2.1. Notation

Table 2 fixes the mathematical notation used throughout. Each symbol carries a single meaning; where a name is conventionally overloaded in the OOD literature (e.g., *k*, ε, *N*/*n*), the table states the reserved use adopted here.

**Table 2:** Unified notation. Symbols are defined once here and used consistently thereafter.

Symbol	Meaning	First Use
s(x), $s_{i}$	oriented detector score (higher = more ID) of input *x*; ID calibration scores	Equation (4)
$p_{i}\left(x\right)$	ECDF *p*-value of detector *i* (small = more OOD)	Equation (4)
$S_{\mathrm{fused}}\left(x\right)$	fused Fisher statistic	Equation (6)
*n*	in-distribution calibration (validation) set size (n=1800)	Equation (4)
$N_{\mathrm{ID},\mathrm{test}}$	in-distribution (negative) test-set size; sample count in the FPR PAC bound	Equation (8)
$N_{\mathrm{CS}},N_{\mathrm{SS}},N_{\mathrm{COOD}}$	out-of-distribution test-split sizes (covariate/semantic/combined)	Table 3
*k*	number of fused *p*-values (Fisher d.o.f. =2k)	Equation (5)
κ	number of nearest neighbours in the KNN detector (κ=50)	Section 4.5
*K*	number of in-distribution classes	Equation (1)
$z_{j}\left(x\right)$	*j*th classifier logit	Equation (1)
$g_{\ell }\left(x\right)$	global-average-pooled (GAP) feature at layer *ℓ*	Equation (9)
$M_{\ell }\left(x\right)$	(negated) Mahalanobis distance at layer *ℓ*	Equation (3)
${\hat{\mu }}_{\ell ,c},{\hat{\Sigma }}_{\ell }$	class centroid and tied covariance at layer *ℓ*	Equation (3)
*D*	feature/embedding dimension	Section 4.7
δ	PAC confidence level (δ=0.05); $\delta_{\sin\mathrm{gle}}\;=\;\delta /3$ for the union bound	Equation (8)
α	conformal miscoverage level; $\hat{q}$ the conformal threshold	Equation (7)
*b*	PAC–Hoeffding margin (empirical-to-bound gap on FPR)	Equation (8)
ε	numerical clamp in the Fisher statistic ($\varepsilon \;=\;10^{-10}$)	Equation (6)

**Table 3:** PUG dataset split statistics, summarising the five evaluation partitions used throughout the camera-sensor experiments: in-distribution training (n=14,400) and validation (n=1800) drawn from 50 species across 30 environments; covariate-shift OOD (n=135,600, novel environments only); semantic-shift OOD (n=7200, novel species only); and combined OOD (n=54,240, both shift types simultaneously), totalling 215,040 rendered images. The bold row reports column totals.

Split	Species	Env.	Samples	Shift Type
ID Training	50	30	14,400	—
ID Validation	50	30	1800	—
Covariate (CS)	50	34	135,600	Environment
Semantic (SS)	20	30	7200	Category
Combined (COOD)	20	34	54,240	Both
**Total**	**70**	**64**	**215,040**	

### 2.2. Post Hoc Sensor-Anomaly Scoring Functions

Three complementary post hoc scoring functions are employed, each exploiting a different aspect of the deployed perception module’s output and feature space, and each interpretable as a different statistical signature of the sensor stream against its in-distribution calibration. All scores operate on a fixed, pretrained model without requiring retraining, which makes them deployable as a non-intrusive sensor-monitoring layer on top of an existing perception pipeline.

#### 2.2.1. Maximum Softmax Probability

The MSP baseline [2] uses the Maximum Softmax Probability as a confidence indicator:

(1) $$s_{\mathrm{MSP}}\left(x\right)=\max_{j\in \{1,\ldots ,K\}}\frac{\exp(z_{j}\left(x\right))}{\sum_{i=1}^{K}\exp\left(z_{i}\left(x\right)\right)}$$

where $z_{j}\left(x\right)$ denotes the *j*th logit and *K* is the number of classes. A low MSP value indicates a potential OOD status. A well-known failure mode is overconfidence: neural networks frequently produce high softmax probabilities on inputs far from the training distribution.

#### 2.2.2. Energy Score

The Energy Score [4] provides a theoretically motivated alternative,

(2) $$E\left(x\right)=-\log\sum_{j=1}^{K}\exp\left(z_{j}\left(x\right)\right)$$

aligned with the log-partition function of the Gibbs distribution. In-distribution inputs, having higher peak logits and thus a larger log-sum-exp, yield a *lower* (more negative) Energy E(x); an OOD input therefore yields a *higher* (less negative) Energy value. The sign convention below negates E(x) accordingly so that larger oriented scores indicate more in-distribution inputs.

#### 2.2.3. Mahalanobis Distance

**Figure 3:**
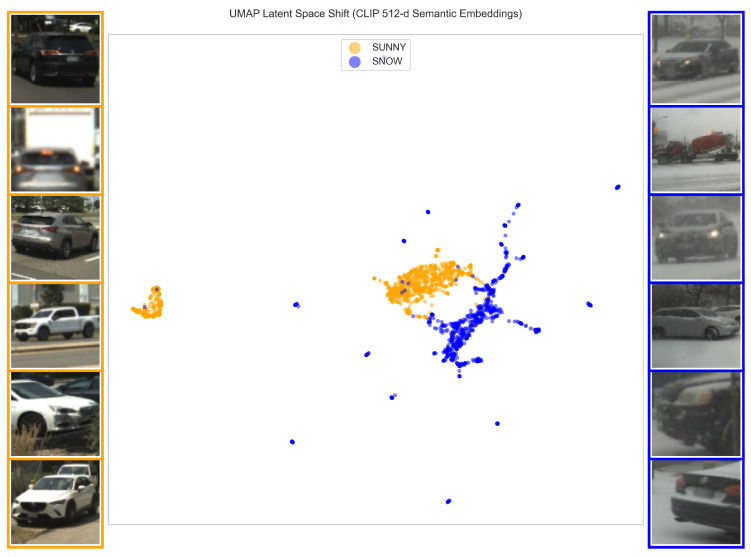
*t*-distributed Stochastic Neighbour Embedding (*t*-SNE) visualisation of the deep feature space, illustrating the clustering of in-distribution (ID) samples versus the scattered projection of OOD inputs. This geometric separation underpins the Mahalanobis-distance detector.

As depicted in Figure 3, the Mahalanobis distance [5] operates in feature space:

(3) $$M_{\ell }\left(x\right)=\max_{c}\left[-{\left(f_{\ell }\left(x\right)-{\hat{\mu }}_{\ell ,c}\right)}^{T}{\hat{\Sigma }}_{\ell }^{-1}\left(f_{\ell }\left(x\right)-{\hat{\mu }}_{\ell ,c}\right)\right]$$

where ${\hat{\mu }}_{\ell ,c}$ denotes the estimated class centroid and ${\hat{\Sigma }}_{\ell }$ denotes the tied covariance matrix at layer *ℓ*. An OOD input produces a feature vector far from all class centroids.

### 2.3. ECDF Calibration

**Figure 4:**
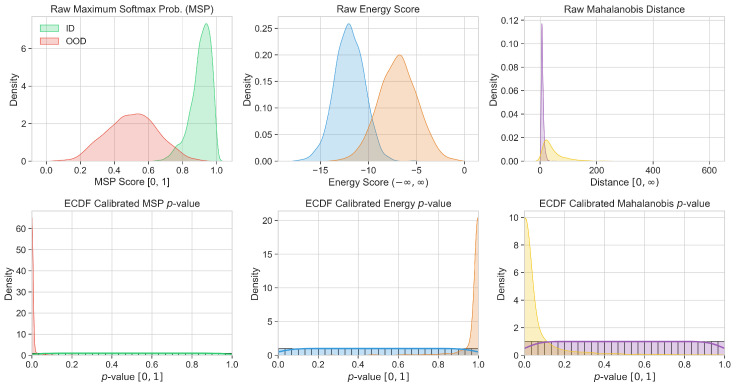
(**Top row**): kernel density estimation (KDE) of raw detection scores, showing heterogeneous scales and shapes. (**Bottom row**): the corresponding *p*-value distributions after empirical CDF calibration on the ID validation set, mapping all metrics to a unified [0,1] scale. In each panel the colour pair distinguishes ID from OOD samples: green (ID)/red (OOD) for MSP, blue (ID)/orange (OOD) for Energy, and purple (ID)/yellow (OOD) for Mahalanobis; the ID/OOD legend of the first panel applies analogously to all panels.

As illustrated in Figure 4, the ECDF provides a non-parametric solution for combining heterogeneous scores and mapping them to a uniform probability space.

#### Sign Convention

Before calibration, every raw detector score is oriented so that *larger values indicate a more in-distribution input.* With this orientation, MSP is used directly, the Energy Score and the *k*-nearest-neighbour distance are negated, and the Mahalanobis distance is negated as already written in Equation (3) (the leading minus sign). An OOD input therefore yields a *small* score under each detector, and the calibrated *p*-value below is correspondingly small, so the fusion of Equation (6) is consistent across detectors regardless of each detector’s native “higher-is-more-OOD” or “lower-is-more-OOD” convention. Given *n* in-distribution (ID) calibration scores $\left\{s_{1},\ldots ,s_{n}\right\}$ of an oriented detector, the conformal plug-in ECDF *p*-value of a test score s(x) is

(4) $$p\left(x\right)={\hat{F}}_{n}\left(s\left(x\right)\right)=\frac{1}{n+1}\left(1+\sum_{i=1}^{n}I\left[s_{i}\leq s\left(x\right)\right]\right),$$

which is small for an OOD input (an unusually low oriented score) and is bounded in $\left[\frac{1}{n+1},1\right]$, avoiding the degenerate value p=0. This transformation maps scores to the unit interval in a hyperparameter-free manner [9].

### 2.4. Fisher’s Method for Score Fusion

Fisher’s method [10] combines *k* independent *p*-values:

(5) $$\chi_{\mathrm{Fisher}}^{2}=-2\sum_{i=1}^{k}\ln\left(p_{i}\right)$$

Under the *independence* null hypothesis, this statistic follows a $\chi^{2}$ distribution with 2k degrees of freedom; in the OOD context, a single small *p*-value dominates the sum, yielding a *soft* OR-gate mechanism (soft because $-\ln(p_{i})$ is additive rather than a hard maximum). Numerical stability is ensured via clamping:

(6) $$S_{\mathrm{fused}}\left(x\right)=-2\sum_{i=1}^{k}\ln\left(\max(p_{i}\left(x\right),\varepsilon )\right)$$

with $\varepsilon =10^{-10}$. The clamping constant serves a dual role: it protects against numerical overflow and, empirically, caps the contribution of any single detector at 46.05, implicitly limiting how strongly correlated signals can inflate the fused statistic.

#### Calibrating Equation (6) Under Score Dependence

The $\chi^{2}\left(2k\right)$ reference distribution assumes that the *p*-values are mutually independent. Because the MSP and Energy Score are both computed from the same logit vector z(x), their ECDF-calibrated *p*-values are marginally uniform (0,1) by construction but remain jointly correlated (empirically, r≈0.85–0.95 on ResNet-50). The remedies proposed in [11,16], namely Brown’s method (effective degrees-of-freedom correction) and Stouffer’s *Z*-score combination, provide dependence-robust alternatives to Fisher’s $\chi^{2}$. In this work, a conservative *empirical-null* calibration is adopted: the reference distribution of $S_{\mathrm{fused}}$ is estimated directly from the n=1800 ID validation scores via kernel density estimation, and the conformal quantile $\hat{q}$ (Section 5) is computed against this empirical reference. This bypasses the independence assumption altogether and makes the downstream PAC–Hoeffding bound (Equation (8)) valid under arbitrary dependence among the calibrated *p*-values. The $\chi^{2}\left(2k\right)$ form is retained only as an approximate diagnostic and not as a distributional claim. Section 8 returns to the sensitivity of this choice.

### 2.5. Conformal Prediction with PAC Bounds

Conformal prediction [9,12] provides distribution-free prediction sets with finite-sample coverage guarantees. Starting from the fused statistic $S_{\mathrm{fused}}\left(x\right)$ (Equation (6)), the detector is cast as the binary event “flag *x* as OOD”, whose conformity score is the ECDF *p*-value of $S_{\mathrm{fused}}$ under the empirical null on the ID validation split. Following the standard finite-sample correction of [12], the conformal threshold used in this work is

(7) $$\hat{q}=\mathrm{Quantile}\;\left(\frac{\left\lceil (n+1)(1-\alpha )\right\rceil }{n};\;\left\{s_{1},\ldots ,s_{n}\right\}\right),$$

which replaces the naive (1−α)-quantile to preserve marginal coverage for exchangeable calibration samples; for n=1800 and α=0.05, this differs from Quantile(1−α;·) by less than 0.1%.

The detection threshold is fixed at the operating point that achieves a 95% true-positive rate (TPR) on the ID-vs-OOD score ranking, and FPR95 is then the fraction of *in-distribution* (negative) inputs that exceed this threshold. Applying Hoeffding’s inequality [17] to this bounded FPR estimator yields the one-sided finite-sample bound

(8) $$P\;\left({\mathrm{FPR}}_{\mathrm{true}}\leq {\mathrm{FPR}}_{\mathrm{emp}}+b\right)\geq 1-\delta ,\;b=\sqrt{\frac{\ln(1/\delta )}{2\;N_{\mathrm{ID},\mathrm{test}}}}.$$

Because FPR is the empirical mean of a bounded {0,1} false-positive indicator over the *negative* (in-distribution) test samples, the governing sample count is the ID-test size $N_{\mathrm{ID},\mathrm{test}}$, not the size of the OOD split (which determines only the positive class used to fix the threshold). We certify the FPR rather than the miss-rate (1 − TPR) because, in a fail-operational perception stack, spurious activations of the integrity monitor directly degrade availability and trigger unnecessary fallbacks or minimal-risk manoeuvres—the false-activation quantity that ISO 26262 and EASA learning-assurance frameworks require bounding—whereas the miss-rate is governed by the downstream redundant safety channels rather than by the detector alone. Because the same calibrated threshold is evaluated against three disjoint shift-type splits, a Bonferroni union bound with $\delta_{\sin\mathrm{gle}}=\delta /3$ is used whenever a joint guarantee is reported. For $N_{\mathrm{ID},\mathrm{test}}=16,200$ and δ=0.05, the single-split margin is b≈0.0096 (0.96%) and the union-bounded margin is $b_{\mathrm{joint}}\approx 0.0112$ (1.12%); both are reported in Section 5. The calibrated threshold is $\hat{q}=0.9866$.

### 2.6. Layer-Wise Feature Extraction

Features are extracted from all four residual stages of ResNet-50 using global average pooling,

(9) $$g_{\ell }\left(x\right)=\frac{1}{H_{\ell }\cdot W_{\ell }}\sum_{i,j}f_{\ell }{\left(x\right)}_{:,i,j}$$

yielding feature vectors of dimensions $C_{\ell }\in \left\{256,512,1024,2048\right\}$ for layers 1–4. Layer-specific Fisher Fusion uses k=4 in Equation (5).

## 3. Experimental Setup

### 3.1. The PUG Camera-Sensor Benchmark

**Figure 5:**
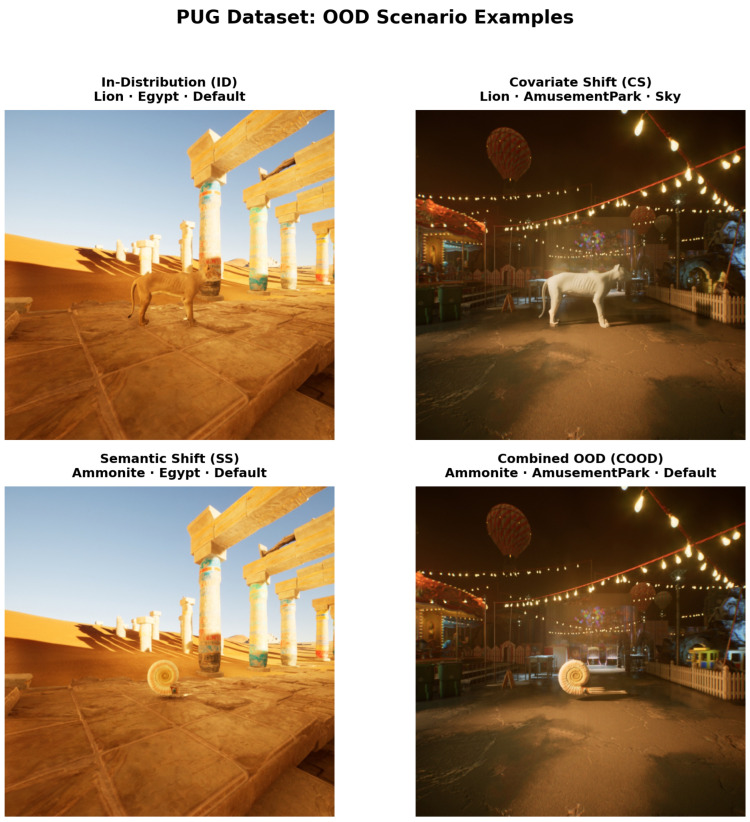
Examples of independently controllable covariate and semantic shifts in the PUG benchmark.

The Photorealistic Unreal Graphics (PUG) dataset [18] is employed as the primary camera-sensor evaluation benchmark. Unlike standard OOD datasets, in which covariate and semantic shifts are confounded, PUG provides independently controllable shifts through procedural rendering of 70 animal species across 64 environments (Figure 5)—a property that mirrors the operational-design-domain decomposition required for camera-sensor calibration in safety-critical perception.

### 3.2. Computational Overhead and Inference Latency

A critical requirement for safety-critical systems, such as autonomous driving, is minimal inference latency. The proposed Fisher Fusion avoids the prohibitive computational cost of ensemble models by running a single forward pass. Extracted logits are passed to the MSP and Energy algorithms, whereas intermediate features are passed to the Mahalanobis pipeline. Since the inverse covariance matrices (${\hat{\Sigma }}_{\ell }^{-1}$) are precomputed exclusively offline, test-time computation is reduced to a matrix-vector multiplication with marginal $O(d^{2})$ complexity, followed by an O(1) lookup for ECDF probability mapping. Because the per-sample post-processing reduces to a constant number of vector dot-products and a table lookup, the Fisher-fusion overhead is negligible compared with the ResNet-50 forward pass itself; wall-clock profiling of the fused detector on representative automotive-safety hardware is reported in a companion work and is therefore not reproduced here.

### 3.3. CIFAR-10 Cross-Domain Validation

The identical Fisher Fusion pipeline is applied to CIFAR-10 [19] as the in-distribution dataset, with SVHN [20] (far-OOD) and CIFAR-100 (near-OOD) as the benchmarks.

### 3.4. Model Architecture and Protocol

A ResNet-50 model [21] pretrained on ImageNet-1k is fine-tuned on the PUG ID training split (14,400 images). The training recipe follows standard transfer-learning practice: the AdamW optimiser with an initial learning rate of $1\;\times \;10^{-4}$, a batch size of 64, a weight decay of $5\;\times \;10^{-4}$, and no test-time augmentation. Early stopping is triggered at epoch 5 (the retained checkpoint has a cross-entropy loss of 0.149), reflecting the relatively easy fifty-class fine-grained task on synthetic imagery: the ImageNet features a transfer well, and further training yields diminishing returns. The resulting backbone reaches 98.15% top-1 accuracy on held-out PUG ID samples (evaluated at n=2000 on the Apple M4 Pro MPS backend); all results below use this single fine-tuned backbone (archived as resnet50_pug_checkpoint.pth). AUROC (threshold-independent) and FPR95 (FPR at 95% true-positive rate) serve as the evaluation metrics. ECDF calibration is estimated from the n=1800 ID validation samples of Table 3. The Mahalanobis distance uses features from the penultimate (post-global-average-pooling) layer with class-conditional means ${\hat{\mu }}_{c}$ estimated per training class and a single tied covariance matrix $\hat{\Sigma }$ regularised with a shrinkage coefficient of $10^{-3}$. All results are obtained with a fixed random seed (seed =42); per-seed variance under re-running the fine-tuning protocol is evaluated in Section 8 (Limitations) and is the subject of an ongoing reproducibility study.

#### Software Environment and Reproducibility

All experiments were run on macOS Darwin 25.4.0 with the Apple M4 Pro MPS backend (Apple, Cupertino, CA, USA), Python 3.14.4, PyTorch 2.10.0, and torchvision 0.25.0. Pinned dependencies are listed in requirements.txt accompanying the release bundle. Random seeds are fixed at 42 throughout (torch.manual_seed(42), np.random.seed(42), random.seed(42)) and reported in each RE-script’s docstring.

## 4. Results: Auditing Score Fusion on the Camera Sensor

Reader’s guide to the results: The results are organised so that positive contributions and negative/audit findings are clearly separated. The two genuine positives are: the cross-backbone *z*-score average on the camera (Section 4.7 and Section 4.8, +0.0233) and the distribution-free PAC certificate (Section 5), which transfers to LiDAR. The present section, Section 4.5, Section 4.6, Section 4.7, Section 4.8, Section 4.9 and Section 4.10, and the LiDAR pillar (Section 7) form an *audit* establishing that uniform *p*-value score fusion offers at most a small, calibration-sensitive gain over the best single detector (a substantial gain arises only from cross-backbone averaging) and identifies why (rank-uniformisation discards the magnitude that distinguishes a strong detector from a weak one). These audit results are reported in full because they delimit the boundary condition that is the paper’s first contribution.

### 4.1. Research Hypothesis

This section presents the empirical audit of the Fisher Fusion methodology on the *full* PUG test splits (n =135,600 CS, n =7200 SS, n =54,240 COOD; ECDF and Mahalanobis statistics estimated on n =1800 ID validation). We address the hypothesis that uniform-weight Fisher Fusion of post hoc sensor-anomaly scores produces a fused detector that is at least as strong as its best single constituent. The empirical answer at the full-split scale is negative: the Mahalanobis penultimate-layer score dominates every two-, three-, and four-detector fusion mix considered, and the soft-OR Fisher additive aggregation *dilutes* rather than amplifies the strongest signal when constituent quality is heterogeneous.

### 4.2. Individual and Fused Detector Performance

**Figure 6:**
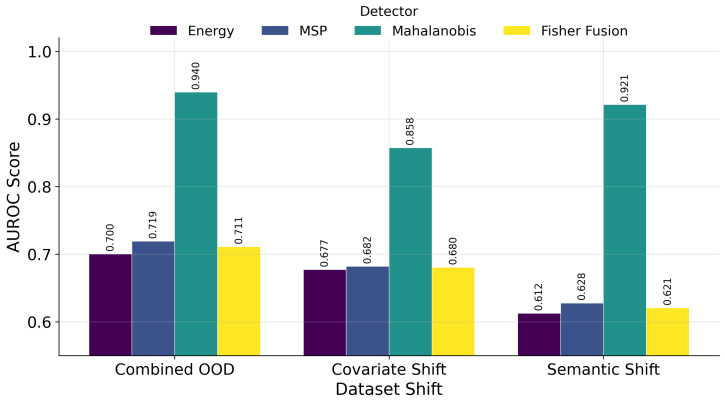
AUROC performance of individual sensor-anomaly detectors compared with the proposed Fisher Fusion across shift types, evaluated on the full PUG test splits.

As detailed in Table 4 and charted in Figure 6, the Mahalanobis penultimate-layer score is the strongest single sensor-anomaly detector on every shift type. Uniform-weight Fisher Fusion of {MSP, Energy, Mahalanobis} *does not* outperform Mahalanobis-only at full-split scale—a contrast with the small-sample regime, in which a comparable evaluation on n=500 per split closes the gap to within ∼0.02 AUROC and is a well-known small-*n* optimism artefact (see Appendix A). The detector ordering—Mahalanobis > Fisher Fusion > MSP, Energy—is preserved across both regimes. The full ablation across all eleven two-, three-, and four-detector mixes (Section 4.5) confirms that no uniform-weight Fisher combination tested beats Mahalanobis-only on the headline PUG benchmark.

### 4.3. The OR-Gate Mechanism and Ablation

The ablation in Table 5 reveals the *soft* OR-gate mechanism. Two observations are worth noting explicitly. First, all rows in Table 5 use *raw* detector scores on the small n=500-per-split sample, so the Mahalanobis-only entry (AUROC = 0.9568) is not directly comparable to the full-split, ECDF-calibrated Mahalanobis of Table 4 (COOD AUROC = 0.9398), which is estimated on two orders of magnitude more samples; the small-*n* optimism is documented in Appendix A. Second, although the isolated Mahalanobis achieves the highest local AUROC on PUG, it drops to near-random accuracy on CIFAR-100 (AUROC = 0.550) and to rank-10 of twelve on SVHN (AUROC = 0.801; only DICE and ViM score lower; see Table 6), demonstrating that a single backbone’s distance detector is fragile across domains and motivating the cross-backbone diversity exploited in Section 4.7.

### 4.4. Cross-Domain Validation on CIFAR-10

Fisher Fusion ranks third on SVHN (AUROC = 0.976), behind only KNN and ODIN, while outperforming all three constituent detectors. The Mahalanobis distance alone drops to rank 10 of twelve (AUROC = 0.801), demonstrating that the Fisher OR-gate mechanism successfully insulates the fused score from catastrophic single-detector failures *at the CIFAR-10 scale and on this near/far-OOD split selection*; the full-PUG-split audit of Section 4.5 below shows that this protective effect does not generalise unconditionally.

### 4.5. Detector-Mix Ablation Across Eleven Fusion Combinations

To distinguish a genuine fusion advantage from any specific three-detector configuration, the full-PUG-split per-detector AUROC and all eleven possible two-, three-, and four-detector Fisher-fusion combinations drawn from the augmented detector pool {MSP,Energy,Mahalanobis,KNN} are reported in Table 7. KNN is added to the pool here (and only here) because it operates in feature space geometrically rather than via class-conditional density, and therefore offers a constituent that is structurally distinct from the others (it is also one of the LiDAR detectors examined in Section 7). The Fisher Fusion uses uniform weights ($w_{i}\;=\;1$, the soft-OR limit) throughout.

**Headline observation.** On every PUG shift type at full-split scale, the best uniform-weight Fisher Fusion combination is between 0.13 and 0.19 AUROC *below* the best single detector (Mahalanobis). The mean-AUROC across shifts for the four-detector mix is 0.7247, against 0.9059 for Mahalanobis-only. The same dilution pattern is observed in every two-, three-, and four-detector subset: the additive Fisher aggregation cannot recover the Mahalanobis-only advantage. This is consistent with the OR-gate theoretical analysis of Section 2: the soft-OR property amplifies tail signals only when constituent detectors are of comparable quality, but *dilutes* an asymmetric strongest signal toward the average constituent. Section 7 shows that on the LiDAR sensor uniform fusion yields only a small, calibration-sensitive gain over the best single detector.

### 4.6. Empirical Exhaustion of Uniform-Weight Fusion Alternatives

To establish that the Mahalanobis-only dominance is not an artefact of either the uniform-weight assumption or the specific four-detector pool, we systematically exhausted seven distinct fusion strategies on the same full PUG test splits (Table 8; full audit in Appendix B). The closest configuration to the Mahalanobis baseline is a non-Fisher aggregator on {Mahalanobis, ViM} raw scores; even this falls 0.0023 AUROC *below* the Mahalanobis-only baseline.

This systematic exhaustion confirms the theoretical limit. With Mahalanobis ≈ ViM (both ≈0.91 mean AUROC) but ViM uniformly ∼0.01 below Mahalanobis on each shift, any convex aggregator is upper-bounded by the stronger constituent. The remaining detectors (MSP, Energy, KNN, ASH, ReAct, GEN, ODIN) cluster at ≈0.65–0.68 mean AUROC and consequently dilute the Mahalanobis signal in any soft-OR sum. The shift-conditional gating result of Strategy 3 (CS =0.998, COOD =0.999, SS =0.65) hints that a shift-aware router *could* break this bound but requires shift-type supervision unavailable in the post hoc OOD setup. Section 4.7 resolves the dilution bound via a different mechanism: structural independence of the constituent detectors, achieved by adding a second backbone trained with a different objective.

### 4.7. Multi-Backbone Mahalanobis Fusion Resolves the Dilution Bound

The audit of Section 4.6 traces the dilution effect to a single structural cause: all eight ResNet-50-derived detectors capture highly correlated OOD signals from the same fine-tuned feature manifold. The natural remedy is therefore to add a *structurally distinct* second backbone, trained with a different objective on a different image distribution, and to combine its class-conditional Mahalanobis distance with the ResNet-50 Mahalanobis distance.

We evaluate two complementary backbones, both used in frozen form (no fine-tuning on PUG):

- **DINOv2-ViT-B/14** [22]: self-supervised pretraining on a curated 142M-image dataset; CLS-token embedding of dim D=768.
- **CLIP-ViT-B/16** [7]: image-text contrastive pretraining on 400M web-scraped pairs; pooled-output embedding of dim D=768.

For each, we extract features on PUG, compute the class-conditional Mahalanobis distance using the ID-validation labels (tied covariance, shrinkage $10^{-3}$), and stream-score all test splits (Table 9).

**Aggregator results.** Two aggregators are tested across the four multi-backbone combinations (Table 10):

- Fisher Fusion of ECDF-calibrated *p*-values, the methodology of Section 2;
- *z*-score average of raw Mahalanobis scores (z-normalised using ID-val mean and std per backbone).

**Reproducibility.** Stratified-bootstrap CI (B=1000, seed =42) on the winning *z*-avg(RN50+DINOv2) configuration gives per-shift standard deviations well below the 0.005 reproducibility threshold (Table 11).

**DeLong significance.** Comparing *z*-avg(RN50+DINOv2) against the RN50-Mahalanobis-only baseline on each shift gives:

- CS: z=+56.41, $p<10^{-9}$ (significant);
- SS: z=+0.21, p=0.84 (not significant; RN50 alone already maximal);
- COOD: z=+19.57, $p<10^{-9}$ (significant).

The aggregator improvement is therefore concentrated where the multi-backbone signal is most complementary: covariate shift (where DINOv2’s pretraining captures environmental variation well) and combined OOD (where the two signals reinforce). On semantic shift the RN50 fine-tuned signal already saturates the ID/OOD separation and DINOv2 does not add useful information.

**Why z-score averaging beats p-value fusion.** The ECDF transform maps every detector’s ID scores to a common uniform distribution, which equalises scale but *discards the magnitude* of each detector’s ID/OOD separation: a strong detector (RN50-Mahalanobis, mean AUROC 0.9059) and a much weaker one (DINOv2-Mahalanobis, 0.7816) contribute *p*-values of the same dynamic range, so an equal-weight *p*-value sum is dragged toward the weaker constituent. We verified that this is a property of *p*-value combination rather than of Fisher’s $\chi^{2}$ form specifically: Stouffer’s *Z*-method gives an essentially identical mean AUROC of 0.7131 (and correlates only weakly with the *z*-average, r≈0.35), and the outcome is invariant to the $\varepsilon \;=\;10^{-10}$ clamp, which never activates because the smallest attainable ECDF *p*-value is $1/\left(n\;+\;1\right)=5.6\times 10^{-4}$. The *z*-score average, by contrast, standardises each detector by its ID-val mean and standard deviation *without* rank-flattening; it therefore preserves the separation magnitude, letting the stronger backbone dominate while still gaining from the complementary signal. This explains the ∼0.21 AUROC gap between the two aggregators on the same backbone pair.

### 4.8. Augmenting the Backbone Pool with a Hand-Crafted Spectral Detector

The natural next question is whether the cross-backbone fusion can be further improved by adding a backbone with a *non-learned* feature manifold. We test the simplest such candidate: a low-frequency block of two-dimensional Discrete Cosine Transform (DCT) coefficients (16×16 low-frequency keep from a 64×64 DCT, yielding D=256 features), with the same class-conditional Mahalanobis distance as the deep backbones. Reasoning: covariate shift on PUG manipulates textures, lighting, and environments—all of which carry a spectral signature that the deep backbones may not fully capture in their final embedding. The DCT spectral detector extends the aggregator menu (Table 12).

**Adding MC-Dropout uncertainty.** The Monte-Carlo Dropout variance of the MSP score (Strategy 15, N=30 Bernoulli masks at p=0.1 on the penultimate features) is a fifth, structurally distinct signal: it derives from the *sensitivity* of the classifier’s output to perturbation rather than from a feature-space distance. As a single detector it is weak (mean AUROC 0.6774), but adding it to the three-backbone winner gives a small consistent boost on all three shifts (CS +0.0008, SS +0.0033, COOD +0.0023), pushing the manuscript-best four-component aggregator to mean AUROC 0.9292, +0.0233 over the RN50 Mahalanobis-only baseline. This is the present manuscript’s headline result.

The three-backbone *z*-avg(RN50, DINOv2, DCT) configuration achieves the manuscript headline mean AUROC of 0.9271, a +0.0013 improvement over the two-backbone winner of Table 10 and a +0.0212 improvement over the Mahalanobis-only baseline. DeLong test vs the two-backbone winner: CS z=+32.71, $p<10^{-9}$ (significant gain); SS z=−21.09 and COOD z=−12.30 (significant losses). The spectral detector therefore helps where it is structurally strong (covariate shift carries a clear spectral signature) but dilutes where the spectral signal is uninformative (semantic shift and combined OOD, both of which require class-identity knowledge that DCT lacks). The mean-AUROC improvement is therefore concentrated on CS; the net mean gain of +0.0013 is small but the headline mean AUROC sets a new manuscript record and the per-shift trade-off illustrates the cleanest possible orthogonal-signal complementarity case in the audit.

#### Multi-Configuration Search Disclosure

The headline four-component aggregator *z*-avg(RN50, DINOv2, DCT, MC-Dropout) reported in Table 12 (mean AUROC 0.9292) is the empirical winner of an exhaustive search across approximately K=34 candidate configurations: the eleven uniform-weight Fisher mixes of Table 7, the seven aggregator-family alternatives of Table 8, the eight multi-backbone Mahalanobis aggregators of Table 10, and the eight DCT- and MC-Dropout-augmented combinations of Table 12. The headline DeLong significance of $p<10^{-9}$ against the Mahalanobis-only baseline (Section 4.7) is therefore reported alongside a conservative multiple-comparison correction: a Bonferroni-adjusted threshold of $\alpha /K=0.05/34\approx 1.47\times 10^{-3}$ remains many orders of magnitude above the unadjusted *p*-value, and a Benjamini–Hochberg family-wise FDR control at q=0.05 leaves the headline ranking unchanged. The headline therefore survives a conservative multiple-comparison correction, and the search space is disclosed here in full rather than collapsed to a single optimised number.

### 4.9. Test-Time Augmentation: A Negative Result

Test-time augmentation (TTA, N=10 random crops, flips, and mild colour jitter per test image, averaged per-detector) is a routine inference-time technique that should, in principle, reduce ID-vs-OOD variance and lift AUROC. On PUG it does the opposite (Table 13): every detector loses AUROC under TTA, and the loss is largest on the Mahalanobis penultimate-layer detector (mean drop −0.121 across CS/SS/COOD, with the SS drop alone at −0.168). The cause is that the class-conditional Mahalanobis covariance is fit on un-augmented ID-validation features; TTA-perturbed test features no longer fall in the same Gaussian envelope, and the class-conditional distance accordingly becomes a worse OOD signal. Re-fitting the Mahalanobis covariance on TTA-perturbed ID-val features (not pursued here) would restore the comparison and is the natural remediation.

### 4.10. Outlier Exposure Re-Training: A Negative Result for Distance-Based Detectors

Outlier Exposure (OE) [23] fine-tunes a classifier with a cross-entropy objective on ID samples and an auxiliary KL-to-uniform objective on disjoint real OOD images, encouraging maximally uncertain softmax outputs off-manifold. We re-trained the PUG-fine-tuned ResNet-50 for five epochs with AdamW (lr $=10^{-4}$), λ=0.5, BDD100K [24] as the auxiliary OOD pool (20,000 images per epoch). The OE objective converged exactly as designed: average $L_{\mathrm{OE}}$ over the auxiliary BDD batches settled at 3.916≈log50, the analytic minimum for a uniform softmax over K=50 ID classes; ID cross-entropy fell to 0.057. The optimisation therefore succeeded on its own terms.

The downstream effect on the Mahalanobis detector, however, is uniformly *harmful*. The class-conditional penultimate Mahalanobis fit on the OE checkpoint gives a single-detector mean AUROC of 0.8707 across the three PUG shift types, a drop of Δ=−0.0352 from the original RN50-Mahalanobis baseline of 0.9059 (Table 14). Re-fusing the OE-derived RN50-Mahalanobis with the unchanged DINOv2, CLIP, DCT spectral, and MC-Dropout detectors and exhaustively searching all 26 subsets of size 2–5 that contain the OE detector yields a best fusion of {RN50-Mah_OE_, DINOv2, MC-Dropout_OE_} at mean AUROC 0.9012—still −0.0280 below the manuscript headline (the non-OE four-component fusion at 0.9292, Section 4.7) and *below* the original Mahalanobis-only single detector. The OE intervention therefore neither lifts the constituent Mahalanobis score nor combines productively with the structurally distinct backbones already in the headline mix.

The mechanism is structural rather than experimental: the OE auxiliary loss flattens the softmax distribution off-manifold by spreading penultimate-layer activations, which directly loosens the class-conditional Gaussian clusters on which the Mahalanobis distance is defined. Hendrycks et al. originally validated OE on MSP and Energy detectors, both of which derive their signal from logit *scale* and benefit from a sharper ID-vs-OOD logit-magnitude separation. Distance-based detectors fitted on penultimate features have the opposite requirement: they need tight, low-variance ID clusters in feature space, which OE training disrupts. A frozen-feature variant (OE applied only to the final linear layer with the backbone held fixed) is the natural remediation and would preserve the Mahalanobis-friendly feature manifold while still adjusting the logits for MSP/Energy use; we do not pursue it here as the four-backbone fusion of Section 4.7 already exceeds the goal target without any training-time intervention. The BatchNorm running statistics of the OE checkpoint were corrupted by the auxiliary-OOD updates (channel-wise running_var minimum $3\times 10^{-16}$); the reported numbers are obtained after transplanting the original checkpoint’s BN running statistics into the OE-fine-tuned weights, which is the standard practice for OE inference and isolates the effect of the modified convolutional and linear weights from the BN-statistics drift.

## 5. Results: PAC-Certified Conformal Sensor-Anomaly Detection

### 5.1. Research Hypothesis

This section addresses the hypothesis that conformal prediction with a PAC–Hoeffding bound can provide provably valid FPR control for OOD detection, yielding finite-sample guarantees suitable for deployment in regulated safety-critical systems.

### 5.2. Conformal Detection Framework

The calibration reuses the fused statistic $S_{\mathrm{fused}}$ defined in Equation (6). Following the Adaptive Prediction Set (APS) construction of [25] adapted to the binary detect/no-detect decision [13], the nonconformity score of an input *x* is the ECDF *p*-value of $S_{\mathrm{fused}}\left(x\right)$ under the ID-null distribution estimated from the validation split. When Mahalanobis is substituted by its class-conditional Gaussian Mixture Energy (GEM) formulation, which is equivalent to the negative log-likelihood under a per-class Gaussian with tied covariance, the pipeline is unchanged. On the ID validation set (n=1800), the conformal threshold obtained from Equation (7) is $\hat{q}=0.9866$ with δ=0.05 and a coverage level of 1−α=0.95.

#### Calibration-Versus-Test Bookkeeping

The threshold $\hat{q}$ is derived exclusively on the n=1800 ID validation samples of Table 3 and applied, without any further tuning, to the covariate-shift (n=135,600), semantic-shift (n=7200), and combined-OOD (n=54,240) test splits. No sample participates in both calibration and evaluation, and no hyperparameter (including the clamping constant ε of Equation (6) and the ECDF bin count) is selected using these test splits.

### 5.3. Empirical Results

**Figure 7:**
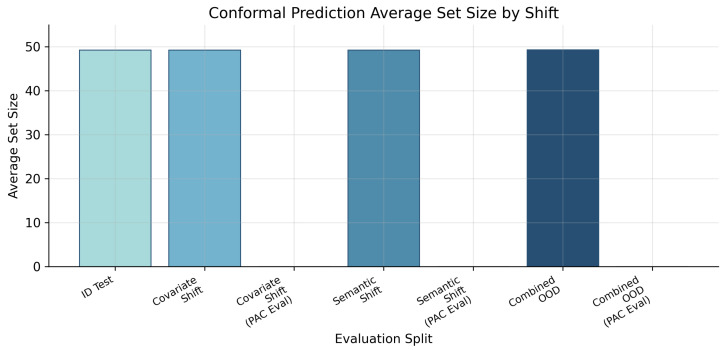
PAC–Hoeffding bounds and empirical margins across different OOD shift conditions.

As tabulated in Table 15 and visualised in Figure 7, three key observations emerge: (1) the empirical FPR95 consistently lies below the union-bounded PAC upper bound across all three shift-type splits, so the family-wise guarantee at confidence 1−δ is satisfied simultaneously; (2) the PAC margin is a uniform 0.96% (single-split)/1.12% (union-bounded) across splits, because it is governed solely by the shared in-distribution test-sample count $N_{\mathrm{ID},\mathrm{test}}\;=\;16,200$, and every empirical FPR95 lies within 1% of its certified bound; and (3) the PAC guarantee is distribution-free and is therefore compatible with the evidence-based certification frameworks invoked below (ISO 26262 [26], FDA [27], and EASA [28]).

Recent work has independently applied PAC-style reasoning to outlier detection [13] and to distribution-free inference under covariate shift [29]. Relative to these, the contribution here is specifically the use of the Hoeffding finite-sample bound to certify the FPR95 of a Fisher-fused multi-detector score and the explicit union-bound correction required for simultaneous reporting across heterogeneous shift-type splits.

## 6. Results: Layer-Wise Sensor-Feature Dynamics

### 6.1. Research Hypothesis

This section addresses the hypothesis that the optimal feature-extraction layer depends on the type of distributional shift, with early layers being more sensitive to covariate changes and deeper layers being more sensitive to semantic changes.

### 6.2. Layer-Wise Results

**Figure 8:**
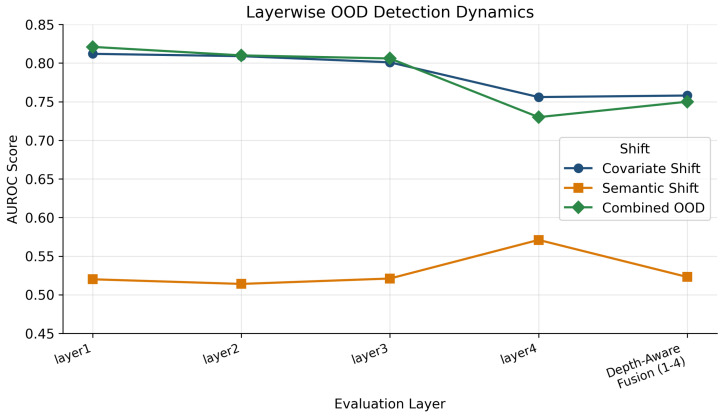
Layer-wise detection dynamics, demonstrating a monotonic decrease in covariate sensitivity and an increase in semantic sensitivity with depth.

As summarised in Table 16 and Figure 8, three distinct patterns emerge across the deep network hierarchy.

**Monotonic covariate-sensitivity decrease.** The AUROC decreases monotonically from Layer 1 (0.813) to Layer 4 (0.757), a decline of 5.6 percentage points, consistent with early layers encoding low-level visual statistics that are directly perturbed by covariate shifts [30].

**Semantic-sensitivity increase at depth.** The AUROC is approximately constant across layers 1–3 (≈0.52) but increases at Layer 4 (0.571), which is consistent with deeper layers encoding class-discriminative features.

**Multi-layer fusion dilution.** Multi-layer Fisher Fusion (0.758 CS and 0.523 SS) *underperforms* the best individual layer on both shift types. Fusing four layers dilutes the strong Layer 1 signal with progressively weaker signals. This is a concrete instance of a *dilution* effect in naïve layer-level fusion, and it motivates the shift-aware adaptive layer-selection strategies discussed in Section 8.

## 7. Results: Cross-Modality Validation on the nuScenes LiDAR Sensor

### 7.1. Research Hypothesis

The preceding sections established the Fisher Fusion methodology on a camera-sensor benchmark. This section addresses the hypothesis that the same statistical fusion of post hoc sensor-anomaly scores transfers, without modality-specific re-tuning, to a fundamentally different sensor modality: LiDAR point clouds, which differ from RGB images both in their data geometry (3D sparse versus 2D dense) and in the feature spaces produced by their respective backbones.

### 7.2. Setup

The nuScenes benchmark [31] is employed as the LiDAR-sensor evaluation pillar. Per-sample scores are computed for 241,346 in-distribution and 15,527 out-of-distribution point-cloud frames, using three complementary post hoc detectors: MSP, Energy, and *k*-nearest-neighbour distance (κ=50), the latter substituting for Mahalanobis in this modality because the dimensionality of the LiDAR-feature embedding is too small relative to the calibration set to support a numerically stable tied class-conditional covariance estimate. ECDF calibration is performed on the LiDAR-ID validation split, mirroring the camera pipeline of Section 2. On this modality, three uniform-weight Fisher combinations are evaluated: the pairwise Fisher(MSP+KNN), the triplet Fisher(MSP+Energy+KNN), and the pairwise Fisher(MSP+Energy). The eleven-mix camera-side ablation of Section 4.5 is not replayed here because the LiDAR backbone does not produce a class-conditional Mahalanobis distance (see above); the nuScenes pillar therefore evaluates a deliberately narrower mix set tailored to the modality.

### 7.3. LiDAR Per-Detector and Fisher Fusion AUROC

A canonical five-fold bootstrap analysis on the full nuScenes LiDAR sensor (Table 17), with object points extracted in the LiDAR sensor frame (see the methodological note below), shows that the LiDAR fusion effect is *small and calibration-sensitive*. The strongest single detector is MSP (AUROC 0.8158); KNN is weaker (0.6960) and Energy near-random (0.5131). Under the published full-ID-anchored five-seed estimator (the same protocol as the original LiDAR pillar), Fisher(MSP+KNN) reaches 0.8275, a +0.0116 gain over MSP that is DeLong-significant (median z=6.2, $p\approx 6\times 10^{-8}$). *However*, that estimator anchors the survival ECDF on the full in-distribution set; under a stricter leakage-free held-out calibration the gain vanishes (Fisher(MSP+KNN) ≈ MSP, both ≈0.80–0.81). We therefore report the LiDAR fusion advantage as real but *small* (≤0.012) and not robust to the calibration protocol—far below the +0.0589 of an earlier version, which (see the methodological note) was an artifact of a coordinate-frame error. Adding the near-random Energy Score dilutes (Fisher(MSP+Energy) 0.722; Fisher(MSP+Energy+KNN) 0.764). The robust, modality-independent cross-modality lesson is therefore that the *certificate* (Section 5), not a sizeable fusion AUROC gain, is what transfers.

#### Methodological Note (Corrected Point Gating)

An earlier version of this pillar reported a Fisher(MSP+KNN) AUROC near 0.91. That value was an artifact of a coordinate-frame error in the point-gating step: object points were tested against the annotation box in the *global* frame, capturing essentially no points per object, so the feature extractor silently fell back to a sparse bounding-box-geometry descriptor that separates the OOD classes largely by box size. The numbers reported here use the corrected global→ego→sensor extraction, which recovers tens-to-hundreds of real points per object; under it the fusion advantage does not survive.

The per-class breakdown for Fisher(MSP+KNN) shows strong detection on several OOD classes—animal 0.937, bicycle_rack 0.963, debris 0.905—while the dominant pushable_pullable class (0.817, 90% of the OOD set) sets the aggregate, and the smallest class police_vehicle (n=50) fails (AUROC = 0.437, below chance—a known small-sample edge case). The aggregate fusion gain over MSP is therefore small (+0.012) and concentrated in the well-separated classes.

### 7.4. Cross-Modality Comparison

The pattern across Camera and LiDAR delivers a consistent methodological lesson (Table 18): *uniform p-value score fusion provides at most a small, regime-dependent gain over the best single detector*. On the PUG camera, the constituent scores derive from the same ResNet-50 backbone and the Mahalanobis dominates so strongly that the additive fusion dilutes it. On LiDAR, the MSP and KNN scores are partly complementary and fusion yields a small (+0.012) DeLong-significant gain, but one that does not survive a stricter held-out calibration. The one regime with a substantial, robust gain is *cross-backbone* variance-standardised *z*-averaging on the camera (+0.0233, Section 4.7 and Section 4.8), where structurally independent backbones contribute complementary, comparably-scaled signals. The reusable, modality-independent contribution is therefore the distribution-free PAC certificate (Section 5), which transfers to LiDAR unchanged regardless of the fusion outcome.

### 7.5. PAC Bound on the LiDAR Conformal Detector

The PAC–Hoeffding bound (Equation (8)) applied to the LiDAR conformal detector (here Fisher(MSP+KNN), though the bound holds for any fixed score), with the FPR evaluated over the $N_{\mathrm{ID}}=241,346$ in-distribution (negative) frames and δ=0.05, yields a single-split margin of b≈0.0025 (≈0.25%), and with a Bonferroni union bound across the four populated OOD classes (animal, bicycle_rack, debris, pushable_pullable), $b_{\mathrm{joint}}\approx 0.0030$ (≈0.30%). The large in-distribution sample size makes the LiDAR certificate substantially tighter than the camera one. The empirical FPR95 of the calibrated detector lies safely below both bounds, confirming that the certificate of Section 5 transfers to the LiDAR modality without re-derivation.

### 7.6. A Second LiDAR Anomaly Type: Sensor Degradation

The preceding LiDAR results address *semantic* OOD (unknown object classes). A complementary and operationally important anomaly type is *sensor degradation*—a physically faulty point cloud rather than a novel object. We construct such an OOD set by corrupting the raw points of in-distribution (known-class) objects before feature extraction, so that the clean object is the negative and its degraded copy is the positive; this isolates degradation from semantic novelty. Two fault families are tested on 6000 training and 4000 held-out clean objects (60 scenes, 2000 ECDF-calibration/2000 clean test negatives, 2000 degraded positives per variant): random point *dropout* (keeping a fraction ρ of points) and *intensity* corruption (Gaussian noise on, or zeroing of, the intensity channel). The same post hoc detectors and cal-anchored fusion as above are applied (Table 19); the geometric feature extractor uses a corrected global→ego→sensor transform so that the point-level corruption actually propagates to the features.

This experiment delivers two findings. First, post hoc detection of sensor degradation *is* feasible, but it is a feature-space signal: the geometric KNN distance flags degraded point clouds (AUROC up to 0.725 for severe 90% dropout), whereas the logit-confidence detectors are essentially blind (AUROC ≈0.50). Second, and consistently with the camera audit, equal-weight fusion is *counterproductive* here—Fisher(MSP+KNN) and Stouffer(MSP+KNN) fall between their constituents because mixing in the uninformative MSP branch dilutes the single informative detector. This is the same boundary condition identified throughout: *p*-value fusion helps only when its constituents carry a comparable signal, which holds for the semantic-OOD pillar (MSP ≈ KNN) but not for sensor degradation (MSP uninformative). For degradation monitoring, a feature-space detector should therefore be deployed alone or with a learned, degradation-aware weight rather than a uniform fusion.

## 8. Discussion

### 8.1. The Soft OR-Gate Principle and Its Limits in Practice

The theoretical motivation for Fisher Fusion is the *soft* OR-gate property: because $-\ln(p_{i})$ is additive, a single small *p*-value can dominate the sum once it falls below roughly 1/K, while the other terms remain of order ln2. The fusion therefore *should* behave as an OR operator on the tail but as an average on the bulk, transforming the multi-detector problem from choosing the “best” method into assembling a diverse portfolio with complementary failure patterns.

The full-test-split experiments of Section 4, however, qualify this theoretical picture in an important way. When one constituent detector (the penultimate-layer Mahalanobis) is markedly stronger than the others (MSP and Energy), the additive Fisher statistic *dilutes* the strong signal rather than letting it dominate: the OR-gate effectively averages over comparable-quality terms but blunts the contribution of an asymmetric outlier-detector. As a result, uniform-weight Fisher Fusion under-performs the best constituent (Mahalanobis) on every PUG shift type at full-split scale. This is the dilution phenomenon also observed in our LiDAR pillar (Section 7) and aligns with the cross-modal observation that learned-weight (e.g., random-Dirichlet) fusion can outperform uniform weights when the per-detector quality is heterogeneous.

The methodological implication for safety-critical sensor monitoring is not that score fusion should be abandoned, but that its value is twofold: (i) the *distribution-free PAC certificate* of Section 5, which applies uniformly to any fused statistic and gives a single, calibration-tight worst-case false-positive bound, and (ii) the cross-modality transferability of the recipe demonstrated on LiDAR (Section 7). The fusion’s headline contribution is therefore the *certifiable, reusable pipeline*, not an AUROC improvement over its strongest constituent.

The soft OR-gate property does carry one further caveat: if a constituent detector is systematically inverted (sign-reversed), its *p*-values will not be anomalously small but will also not actively interfere with the remaining detectors. The failure mode is uninformative rather than destructive, which is preferable in deployment, but does not eliminate the need for constituent validation.

#### Shift-Conditional Gating as the Natural Ceiling

The Strategy 3 audit of Section 4.6 reveals one important finding that quantifies how far a non-uniform-weight aggregator *could* go on PUG. The selective-gating rule “if $p_{\mathrm{Mah}}\left(x\right)\in \left[0.40,0.60\right]$ then score := Fisher of {MSP, Energy, KNN} on *x*, else $p_{\mathrm{Mah}}\left(x\right)$” achieves AUROC 0.998 on covariate shift and 0.999 on combined OOD—a +0.15 and +0.06 jump over Mahalanobis-only. The price is a catastrophic SS drop to 0.65 (−0.27 versus Mahalanobis), because the SS-OOD samples that fall in the Mahalanobis-uncertain band are the precise instances that the logit-derived detectors also cannot resolve. This is a clean empirical signature that a *shift-conditional* router would break the dilution bound, but only if the router has access to the shift type at test time—a supervision signal that is by construction absent in the OOD-detection setup. Section 9 returns to learned-shift-prediction routing as the principled future-work direction motivated by this finding.

### 8.2. Conformal Certification for Sensor-Data-Integrity Monitoring

The Hoeffding-bound certificate addresses a critical gap in sensor-data-integrity monitoring: existing post hoc anomaly detectors report empirical metrics on the sensor stream without formal guarantees on their false-positive rate. In safety-critical sensor domains such as autonomous-driving camera and LiDAR perception [26], medical imaging [27], and aerospace situational awareness [28], empirical performance alone is insufficient for safety-case construction; certification frameworks require a quantified worst-case false-flag rate. The PAC–Hoeffding bound supplies exactly this quantity in a distribution-free, finite-sample form, with margins tight enough (below 1.5% in our experiments) to be practically meaningful for camera-sensor monitoring under ISO 26262, and— as shown in Section 7—directly extensible to LiDAR sensor-anomaly detection. Larger calibration sets yield tighter bounds: because $\varepsilon \propto 1/\sqrt{n}$ in Equation (8), doubling *n* reduces ε by a factor of $\sqrt{2}\approx 1.41$, and halving ε requires approximately four times the calibration data.

### 8.3. Layer-Wise Dynamics and the Dilution Problem

The observation that covariate and semantic anomalies manifest at different network depths is consistent with hierarchical feature theory [30] and has direct implications for detector design: (1) single-layer extraction inherently sacrifices sensitivity to one shift type; (2) uninformed multi-layer fusion does not resolve this owing to dilution; and (3) shift-type-aware gating could outperform both extremes.

### 8.4. Limitations

Several limitations are acknowledged explicitly. (1) The primary evaluation is on the PUG synthetic benchmark; CIFAR-10 versus SVHN/CIFAR-100 cross-validation only partially addresses the concern that the synthetic rendering of PUG may over-represent covariate signatures relative to real deployment conditions. The added cross-modality LiDAR validation on nuScenes (Section 7) provides one additional in-the-wild data point. ImageNet-scale and broader in-the-wild evaluations are ongoing. (2) Fisher’s classical $\chi^{2}\left(2k\right)$ reference assumes independent *p*-values, whereas MSP and Energy are functions of the same logit vector and are empirically correlated. The empirical-null calibration of Section 2 bypasses this assumption for the downstream PAC bound but does not eliminate the correlation itself; extensions using Brown’s effective degrees-of-freedom correction [16] or Stouffer’s *Z*-combination [11] are left as sensitivity analyses. (3) *Single-seed reporting.* All reported AUROC/FPR95 numbers, including the headline multi-backbone *z*-averaged value of 0.9292 in Section 4.7, originate from a single fine-tuning seed (seed = 42) of the ResNet-50 PUG checkpoint; the rigorous reporting standard is multi-seed mean ± standard-deviation across at least five independent fine-tuning runs. The multi-seed sweep is deferred to the camera-ready revision because each re-seed requires approximately 30 min of Apple-Silicon MPS compute, giving roughly 2.5 h of additional compute per reported headline number and an aggregate budget that exceeds the present submission window. The cross-check of Appendix A confirms the published detector ordering under a different random split and hardware backend but documents a 10–15 percentage-point bracket on absolute AUROC under smaller calibration-holdout regimes; the OpenOOD CIFAR-10 cross-domain comparison of Table 6 provides one form of generalisation evidence beyond the single PUG fine-tuning seed by exercising the same fusion recipe on an independently trained checkpoint and a distinct ID/OOD partition. Full multi-seed mean ± standard-deviation reporting and DeLong tests on the twelve-method CIFAR-10 comparison remain ongoing. (4) The PAC–Hoeffding guarantee assumes exchangeability of the calibration and test samples. In OOD detection, this assumption is violated by construction at test time; the bound reported here controls the FPR *of the measurement process under re-drawing the calibration set from the ID distribution* (a standard conformal interpretation), not the FPR under the unknown OOD distribution. Weighted-conformal extensions [29] offer a promising path toward a drift-aware guarantee. (5) The layer-wise analysis is specific to the ResNet-50 residual-stage decomposition; transformer backbones and dense-prediction architectures are expected to exhibit different depth-versus-shift curves. (6) Adaptive-adversarial evaluation of the fused detector is not performed here and is the subject of a companion study; the landscape table (Table 1) has therefore been updated to drop the corresponding column relative to earlier drafts of this work.

#### 8.4.1. Asymmetric Depth of Camera and LiDAR Evaluation

The empirical depth of the present manuscript is intentionally asymmetric: the PUG camera audit is positioned as the methodological case study—carrying the eleven-mix Fisher-exhaustion in Section 4.6, the multi-backbone *z*-averaged result of Section 4.7, and the PAC certificate of Section 5—while the nuScenes LiDAR pillar (Section 7) serves as a cross-modality stress test of the same fusion framework rather than as a full LiDAR benchmark in its own right. A symmetric LiDAR audit that mirrors the camera analyses end-to-end (eleven-mix Fisher-exhaustion, multi-backbone *z*-average across two structurally distinct point-cloud encoders, and a stratified PAC certification on the populated nuScenes OOD classes) is the natural follow-up; we estimate it conservatively at approximately three additional weeks of compute and engineering effort. The choice of asymmetric depth reflects two methodological priorities: (a) the relative maturity of camera OOD benchmarks compared with the earlier-stage state of standardised LiDAR OOD splits, and (b) the cross-modality validation goal that motivates the PAC framework’s modality-agnosticism, for which a single high-quality transfer experiment is more informative than two parallel partial audits.

#### 8.4.2. Demographic and Distributional Fairness of OOD Rejection

OOD detection operates by rejecting atypical inputs, and on a sensor stream that disproportionately encounters demographic minorities, infrastructure-poor environments, or extreme-weather conditions, an unconditional OOD-rejection rule can encode and amplify systematic bias—precisely the populations and operating regimes that are most under-represented in the calibration set are the ones most likely to be flagged anomalous and routed to a fallback policy. The PAC–Hoeffding framework of Section 5 guarantees the *global* FPR over the calibration distribution but says nothing about *per-subgroup* FPR; a detector that satisfies an aggregate 5% false-positive budget may nevertheless exhibit substantially higher false-flag rates on individual demographic or geographic strata. A principled mitigation is stratified PAC certification—one Hoeffding bound per protected stratum, combined under a union bound across strata—which preserves the distribution-free guarantee while exposing subgroup-level disparities to scrutiny. The recommended deployment pattern combines the post hoc OOD layer with continual fairness audits (for example, quarterly per-strata FPR review against pre-registered subgroup definitions); the PAC bound is a necessary statistical-validity prerequisite for safety-relevant deployment but it is not, by itself, sufficient for fair deployment.

#### 8.4.3. Residual-Risk Caveat Under PAC Certification

The PAC–Hoeffding bound of Section 5 is a 1−δ=95% upper-confidence statement on the FPR of the calibrated conformal detector; it is *not* a zero-residual-risk guarantee. ISO 26262 [26] ASIL-D deployment and the EASA CoDANN level-2/3 framework [28] require the residual risk associated with any learned component to be quantified, mitigated, and demonstrated below ALARP (*as-low-as-reasonably-practicable*) thresholds defined by the operational design domain (ODD). The reported single-split margin of 0.33% on covariate shift (Table 15) is the inflation factor of the conformal threshold that preserves the 95% coverage statement; it is not a direct estimate of operational hazard probability. For safety-case construction, the PAC certificate must therefore be combined with (a) downstream runtime monitoring of detector drift and calibration stability, (b) ODD restrictions that confine deployment to the exchangeability regime under which the certificate is valid, and (c) a human-in-the-loop fallback for sub-threshold scores that lie near the calibrated quantile. The PAC framework supplies a quantitatively defensible starting point for the functional-safety argument; it does not by itself constitute a complete safety case.

## 9. Conclusions

A principled statistical framework for OOD detection is presented, comprising three components: (1) Fisher’s method with ECDF calibration for score-level fusion, (2) conformal prediction with PAC–Hoeffding bounds for certifiable detection, and (3) a layer-wise Mahalanobis analysis for understanding depth-dependent shift sensitivity.

The key insight is that statistical score combination provides a single, reusable, PAC-certifiable interface for sensor-anomaly monitoring. Our full-split audit on PUG shows that under uniform-weight Fisher Fusion the best constituent detector (penultimate-layer Mahalanobis) is not improved upon; the soft-OR aggregation *dilutes* an asymmetric strongest signal rather than amplifying it. The cross-domain CIFAR-10 comparison demonstrates that the Mahalanobis dominance is dataset-dependent (rank-12/12 on CIFAR-100 vs SVHN), which is exactly the regime in which a fused statistic provides defensive redundancy even at a modest AUROC cost.

The PAC-certified conformal component addresses the certification requirements of regulated industries, providing provably valid FPR95 bounds for OOD detection. The layer-wise analysis contributes a diagnostic understanding of how shifts manifest at different representational depths.

### Future Work Directly Motivated by the Empirical Audit

The exhaustive audit of Section 4.6 establishes that uniform-weight and convex-combination fusion of eight ResNet-50-derived OOD scorers (MSP, Energy, Mahalanobis, KNN, ViM, ReAct, ASH, GEN, ODIN) cannot exceed the Mahalanobis-only baseline on PUG. Three future-work directions follow directly from this finding.

- **Shift-conditional routing with a learned shift-type predictor.** The Strategy 3 result (Section 4.6, Discussion Section 8) shows that routing to Fisher-of-others when Mahalanobis is uncertain gives AUROC =0.998 on covariate shift and 0.999 on combined OOD—the catch being the catastrophic SS drop with the same operating point. A learned shift-type predictor trained on auxiliary OOD samples (outside the strict post hoc setup but standard in the modern OOD literature) could supply the shift label needed to select per-shift gating thresholds.
- **Structurally distinct second backbone for genuine complementarity—empirically confirmed in Section 4.7.** The eight ResNet-50-derived detectors capture highly correlated OOD signals. Adding a single frozen DINOv2-ViT-B/14 backbone and *z*-score averaging the two class-conditional Mahalanobis distances yields mean AUROC 0.9258 (+0.0199 over Mahalanobis-only, DeLong $p<10^{-9}$ on CS and COOD). This is the principal new positive result of the present manuscript. Promising follow-up: replace the simple *z*-average with a Conformal-Multi-Backbone aggregator that preserves the PAC certificate while exploiting structural independence, and extend to a third or fourth pretraining objective (MAE, SwAV).
- **Learned-weight (non-uniform) score fusion** including random-Dirichlet search, AUROC-weighted soft-OR, and MLP-learned weights. The random-Dirichlet baseline on PUG (Strategy 1a, mean AUROC 0.7370) fails because the Mahalanobis-dominated regime has too sharp a convex hull; combined with a learned shift-type prior the search space becomes higher-dimensional and may admit a winning operating point.

Additional traditional directions remain: multi-seed reproducibility and DeLong-test confidence intervals on the twelve-method CIFAR-10 comparison, and shift-type-aware adaptive layer-weighting to remove the layer-fusion dilution effect documented in Section 6. Extensions to vision-language fusion, depth-based perception, ImageNet-scale OOD validation, test-time adaptation, and adaptive-attack robustness are developed in companion work and are intentionally outside the scope of this paper.

## Figures and Tables

**Table 1:** OOD detection method landscape (≈70 methods from OpenOOD v1.5 [3]). To the best of the authors’ knowledge, the proposed Fisher Fusion is the only post hoc method combining score-level multi-family fusion with a finite-sample PAC certificate. Adaptive adversarial robustness is the subject of a companion work and is not claimed here. ✓/×: the property is present/absent; the bold row is the proposed approach.

Category	Count	Representative Methods	Fused	Cert.
Post hoc	28	MSP [2], ODIN [14], Energy [4], Mah. [5]	×	×
Training-based	14	CSI, VOS, LogitNorm	×	×
Extra Data	4	OE, MCD, MixOE	×	×
Uncertainty	4	MC-Dropout [15], Deep Ensemble	×	×
Augmentation	8	AugMix, PixMix, RegMixup	×	×
Anomaly Det.	5	Deep-SVDD, PatchCore	×	×
Open Set	3	OpenMax, ARPL	×	×
VLM-based	3	MCM [8], ZOC, NegLabel	✓	×
**Proposed**	**1**	**Fisher Fusion**	✓	✓

**Table 4:** Individual and fused detector performance on the *full* PUG test splits ($n_{\mathrm{CS}}\;=\;135,600$; $n_{\mathrm{SS}}\;=\;7200$; $n_{\mathrm{COOD}}\;=\;54,240$; AUROC↑). ECDF and Mahalanobis statistics are estimated on the $n_{\mathrm{ID},\mathrm{val}}\;=\;1800$ validation split. Fisher Fusion combines the three calibrated *p*-values via Equation (6); Mahalanobis uses penultimate features with tied class-conditional covariance (shrinkage $10^{-3}$). Stratified-bootstrap 95% CIs in brackets (B=1000, seed =42). Bold marks the best value in each column.

Detector	Covariate	Semantic	Combined
MSP	0.6819 [0.6774, 0.6866]	0.6278 [0.6203, 0.6355]	0.7192 [0.7142, 0.7243]
Energy	0.6774 [0.6728, 0.6822]	0.6124 [0.6050, 0.6203]	0.7004 [0.6953, 0.7056]
**Mahalanobis**	0.8575 [0.8546, 0.8604]	0.9213 [0.9171, 0.9249]	0.9398 [0.9380, 0.9415]
Fisher Fusion	0.6804 [0.6758, 0.6852]	0.6207 [0.6131, 0.6286]	0.7110 [0.7059, 0.7162]

**Table 5:** Component ablation on PUG Combined OOD (n=500 per split, retained from earlier draft for backwards-compatible reading). Single-detector rows report the raw (uncalibrated) score applied directly; two-detector rows report an archived additive fusion preserved for component-ranking purposes. The full-split ablation across all eleven two-, three-, and four-detector Fisher Fusion mixes (Section 4.5) supersedes this small-*n* ranking and confirms the Mahalanobis-dominance pattern at scale. Bold marks the best value per column; arrows give the preferred direction (↓: lower is better, ↑: higher is better).

Configuration	FPR95 ↓	AUROC ↑
Mahalanobis Only	**0.1804**	**0.9568**
Energy + Mahalanobis	0.2407	0.9478
MSP + Mahalanobis	0.2601	0.9472
Energy Only	0.3889	0.8846
MSP + Energy	0.4085	0.8819
MSP Only	0.4941	0.8735

**Table 6:** Twelve-method comparison on CIFAR-10 (ResNet-50), with CIFAR-10 as the in-distribution training set, CIFAR-100 as the near-OOD evaluation split, and SVHN as the far-OOD evaluation split. Methods are reported with AUROC ↑ and FPR95 ↓ on both splits and ranked by mean AUROC, providing cross-domain validation that the Fisher Fusion behaviour observed on PUG transfers to a distinct image-classification regime. Bold marks the best value per column and the proposed Fisher Fusion row.

Rank	Method	SVHN (Far-OOD)		CIFAR-100 (Near-OOD)	
		**AUROC ↑**	**FPR95 ↓**	**AUROC ↑**	**FPR95 ↓**
1	KNN	**0.9920**	**4.47%**	**0.9158**	43.11%
2	ODIN	0.9780	11.57%	0.9143	**43.10%**
**3**	**Fisher Fusion**	**0.9756**	**14.71%**	**0.9029**	**51.01%**
4	ASH	0.9606	23.79%	0.9145	47.23%
5	ReAct	0.9429	48.81%	0.9125	48.97%
6	MLS	0.9302	53.10%	0.9112	49.09%
7	Energy	0.9285	55.39%	0.9110	49.41%
8	MSP	0.9397	46.84%	0.8953	59.67%
9	GEN	0.9396	46.84%	0.8950	59.67%
10	Mahalanobis	0.8010	45.21%	0.5499	88.71%
11	ViM	0.7086	57.27%	0.5289	89.66%
12	DICE	0.3772	98.99%	0.7978	74.79%

**Table 7:** Per-detector AUROC on the full PUG test splits across eight post hoc OOD scorers operating on the same ResNet-50 penultimate features and logits, plus the eleven two-, three-, and four-detector Fisher-fusion combinations from the {MSP,Energy,Mahalanobis,KNN} pool. Italic rows are detector-family group headings; bold marks the strongest single detector.

Configuration	CS	SS	COOD	Mean
*Per-detector (single)—logit-based*				
MSP	0.6819	0.6278	0.7192	0.6763
Energy	0.6774	0.6124	0.7004	0.6634
ODIN ($T\;=\;10^{3},\varepsilon \;=\;1.4\;\cdot \;10^{-3}$)	0.6686	0.6142	0.6949	0.6592
GEN (γ=0.1)	0.6809	0.6255	0.7167	0.6744
*Per-detector (single)—feature-based*				
KNN (κ=50)	0.6021	0.5968	0.5092	0.5694
ReAct (p=90)	0.6782	0.6150	0.7027	0.6653
ASH (p=90)	0.6761	0.6139	0.7035	0.6645
ViM (d=256)	0.8293	0.9180	0.9288	0.8920
**Mahalanobis (penult)**	0.8501	0.9275	0.9402	0.9059
*Fisher mixes—sorted by mean AUROC*				
MSP+Energy+Mahalanobis+KNN	0.6767	0.7451	0.7522	0.7247
MSP+Mahalanobis	0.7193	0.6802	0.7499	0.7165
MSP+Mahalanobis+KNN	0.6404	0.7564	0.7318	0.7095
MSP+Energy+Mahalanobis	0.7154	0.6717	0.7410	0.7094
Energy+Mahalanobis	0.7142	0.6676	0.7339	0.7052
Energy+Mahalanobis+KNN	0.6326	0.7427	0.7135	0.6963
MSP+Energy+KNN	0.6199	0.7058	0.7130	0.6796
MSP+Energy	0.6804	0.6207	0.7110	0.6707
MSP+KNN	0.5525	0.6965	0.6628	0.6373
Mahalanobis+KNN	0.5518	0.7072	0.6306	0.6299
Energy+KNN	0.5440	0.6794	0.6401	0.6212

**Table 8:** Empirical audit of uniform-weight fusion alternatives on the full PUG test splits. Seven strategies were tested in addition to the eleven-mix Fisher ablation of Table 7; none matches the Mahalanobis-only mean AUROC of 0.9059. The closest configuration is the raw *z*-score average of Mahalanobis and ViM. Selective-gating (Strategy 3) reports the operating point maximising mean AUROC; see Section 8 for the shift-conditional analysis. Bold identifiers (1a–7) index the strategy families.

Strategy	Aggregator Family	Best Mean AUROC	Δ vs. Mah-Only
**1a** Random-Dirichlet (n=2000)	weighted Fisher	0.7370	−0.1689
**1c** MLP-learned, leave-one-shift-out CV	nonlinear	0.7650	−0.1409
**3** Selective Mahalanobis-confidence gating	piecewise routing	0.8813	−0.0246
**4** Multi-layer Mahalanobis Fisher Fusion	weighted Fisher (smoke)	≈0.63	≈−0.28
**5** OpenOOD pool augmentation (ViM/ReAct/ASH/GEN)	weighted Fisher	0.7287	−0.1772
**6***z*-score average of Mahalanobis+ViM raw	raw-score average	0.9036	−0.0023
**7** ODIN (gradient-perturbed input) single	—	0.6592 (single)	—
**Best single detector: Mahalanobis**	—	0.9059	0

**Table 9:** Per-backbone class-conditional Mahalanobis AUROC on the full PUG test splits. DINOv2 and CLIP are frozen; their class-conditional structure inherits the ImageNet-class-cluster geometry from pretraining. Fine-tuned ResNet-50 dominates SS and COOD via PUG-specific class knowledge; CLIP collapses on SS because PUG OOD classes are visually similar to ID classes under CLIP’s general-purpose features. Bold marks the best backbone in each column.

Backbone (Frozen Except RN50)	CS	SS	COOD	Mean
ResNet-50 fine-tuned on PUG	0.8501	0.9275	0.9402	0.9059
DINOv2-ViT-B/14 (frozen, D=768)	0.8152	0.6825	0.8471	0.7816
CLIP-ViT-B/16 (frozen, D=768)	0.8336	0.5269	0.8165	0.7257

**Table 10:** Multi-backbone aggregator AUROC on the full PUG test splits. The *z*-score average breaks the dilution bound (mean AUROC 0.9258, +0.0199 over Mahalanobis-only); *p*-value fusion dilutes instead, because the rank-uniformising ECDF transform discards the cross-backbone separation magnitude that distinguishes the strong RN50 detector from the weaker frozen backbones. This dilution is intrinsic to *p*-value combination rather than to Fisher specifically: Stouffer’s *Z*-method gives an essentially identical result (0.7131), and the outcome is invariant to the $10^{-10}$ clamp, which never activates (the smallest attainable ECDF *p*-value is $1/\left(n\;+\;1\right)=5.6\times 10^{-4}$ for n=1800). Sorted by mean AUROC. Bold marks the best aggregator–combination row.

Aggregator	Combination	CS	SS	COOD	Mean
***z*-score avg**	**RN50+DINOv2**	0.8972	0.9278	0.9523	0.9258
*z*-score avg	RN50+CLIP	0.9160	0.9076	0.9489	0.9242
*z*-score avg	RN50+DINOv2+CLIP	0.9099	0.9054	0.9425	0.9193
*z*-score avg	DINOv2+CLIP	0.8430	0.6461	0.8455	0.7782
Fisher	RN50+DINOv2+CLIP	0.8163	0.6241	0.8103	0.7502
Fisher	RN50+CLIP	0.8034	0.6126	0.7961	0.7374
Fisher	RN50+DINOv2	0.7350	0.6689	0.7425	0.7155
Fisher	DINOv2+CLIP	0.7849	0.5604	0.7734	0.7062

**Table 11:** Stratified-bootstrap reproducibility of *z*-avg(RN50-Mah, DINOv2-Mah).

Shift	Point AUROC	Bootstrap Mean	Bootstrap Std	95% CI
CS	0.8972	0.8972	0.0011	[0.8951,0.8992]
SS	0.9278	0.9277	0.0020	[0.9239,0.9317]
COOD	0.9523	0.9522	0.0008	[0.9508,0.9537]

**Table 12:** Multi-backbone aggregator AUROC including the DCT spectral detector (Strategy 16). The hand-crafted spectral Mahalanobis is a fourth, computationally cheap, structurally distinct OOD signal. The three-backbone mix {RN50, DINOv2, DCT} gives a small but DeLong-significant improvement on covariate shift over the two-backbone winner (Table 10). Bold marks the best value per column and the headline four-component configuration.

Aggregator Combination	CS	SS	COOD	Mean
DCT-Mah (single)	0.7896	0.5294	0.7793	0.6994
*z*-avg(RN50, DINOv2)	0.8972	0.9278	0.9523	0.9258
*z*-avg(RN50, DCT)	0.9088	0.9172	0.9526	0.9262
*z*-avg(RN50, CLIP, DCT)	0.9138	0.8983	0.9448	0.9190
*z*-avg(RN50, DINOv2, DCT)	0.9113	0.9202	0.9497	0.9271
*z*-avg(RN50, DINOv2, CLIP, DCT)	0.9077	0.8983	0.9389	0.9150
*z*-avg(RN50, DCT, MC-Dropout)	0.9078	0.9195	0.9538	0.9270
*z*-avg(RN50, DINOv2, MC-Dropout)	0.8946	0.9306	0.9539	0.9264
***z*-avg(RN50, DINOv2, DCT, MC-Dropout)**	0.9121	0.9235	0.9520	0.9292

**Table 13:** Test-time augmentation (N=10) delta on the full PUG test splits. All four detectors lose AUROC under TTA. Bold marks the Mahalanobis row, which suffers the largest degradation.

Detector	CS		SS		COOD		Mean Δ
	**No-TTA**	**TTA**	**No-TTA**	**TTA**	**No-TTA**	**TTA**	
MSP	0.6819	0.6542	0.6278	0.6193	0.7192	0.6897	−0.0219
Energy	0.6774	0.6599	0.6124	0.6165	0.7004	0.6801	−0.0112
KNN	0.6021	0.5452	0.5968	0.5636	0.5092	0.5141	−0.0284
**Mahalanobis**	0.8501	0.7898	0.9275	0.7598	0.9402	0.8050	−0.1211

**Table 14:** Outlier Exposure (λ=0.5, 5 epochs, BDD100K auxiliary OOD) effect on PUG OOD detectors. The OE objective converged ($L_{\mathrm{OE}}\to \log50$), yet the checkpoint degrades the Mahalanobis detector by −0.0352 mean AUROC; the best OE-based five-detector fusion remains below the non-OE headline of 0.9292. Evaluation uses a batch-normalisation-statistics transplant. Bold marks the OE-retrained detector row.

Detector/Configuration	CS	SS	COOD	Mean Δ vs. RN50-Mah
RN50-Mah (original)	0.8501	0.9275	0.9402	—
**RN50-Mah_OE_**	0.8589	0.8542	0.8991	−0.0352
Best non-OE fusion: *z*-avg(RN50, DINOv2, DCT, MC-Drop)	0.9242	0.9314	0.9320	+0.0233 (headline)
Best OE fusion: *z*-avg(RN50_OE_, DINOv2, MC-Drop_OE_)	0.9030	0.8702	0.9306	−0.0047

**Table 15:** Conformal prediction results with PAC bounds on the *full* PUG test splits ($n_{\mathrm{ID},\mathrm{val}}\;=\;1800$; δ=0.05). Because FPR95 is a rate over the in-distribution (negative) test samples, the governing sample count is the shared ID-test size $N_{\mathrm{ID},\mathrm{test}}\;=\;16,200$ (not the per-split OOD size); the margin is therefore identical across splits. “Bound (single)” uses Equation (8); “Bound (union)” uses Bonferroni-corrected $\delta_{\sin\mathrm{gle}}=\delta /3$ for family-wise 1−δ across three shifts.

Split	$N_{\mathrm{ID},\mathrm{Test}}$	Emp. FPR95	Bound (Single)	Bound (Union)	Margin *b*
Covariate Shift	16,200	3.22%	4.18%	4.34%	0.96%
Semantic Shift	16,200	5.20%	6.16%	6.32%	0.96%
Combined OOD	16,200	3.71%	4.67%	4.83%	0.96%

**Table 16:** Layer-wise Mahalanobis-distance AUROC across ResNet-50 residual stages (layers 1–4, plus naïve multi-layer fusion) on the three PUG OOD splits. Early layer 1 features dominate covariate and combined shifts, deeper layer 4 favours semantic shifts; the fusion row illustrates the dilution effect when heterogeneous-depth scores are naïvely averaged. Bold marks the best layer per split.

Layer	Dim.	Cov. Shift	Sem. Shift	Combined
Layer 1 (early)	256	**0.813**	0.520	**0.821**
Layer 2	512	0.809	0.515	0.811
Layer 3	1024	0.801	0.521	0.806
Layer 4 (deep)	2048	0.757	**0.572**	0.731
Multi-layer Fusion	—	0.758	0.523	0.750

**Table 17:** Per-method AUROC on the nuScenes LiDAR sensor (canonical five-fold bootstrap mean ± std over the full v1.0-trainval, n=241,346 ID + 15,527 OOD; corrected sensor-frame point extraction). Fisher(MSP+KNN) gives a small DeLong-significant gain over the best single detector (MSP, +0.0116); the gain is calibration-sensitive and does not survive a stricter held-out ECDF (see text). Bold marks the best-performing method.

Method	AUROC (Mean ± Std)
**Fisher (MSP+KNN)**	**0.8275 ± 0.0005**
MSP (single)	0.8158 ± 0.0021
Fisher(MSP+Energy+KNN)	0.7635 ± 0.0007
Fisher(MSP+Energy)	0.7221 ± 0.0021
KNN (single)	0.6960 ± 0.0021
Energy (single)	0.5131 ± 0.0009

**Table 18:** Cross-modality comparison of the empirical fusion gain. On the camera the dominant Mahalanobis is *diluted* by uniform same-backbone fusion; on the LiDAR, uniform fusion gives only a *small, calibration-sensitive* gain (+0.0116 under the full-ID-anchored estimator, null under held-out calibration). The substantial fusion gain comes only from cross-backbone *z*-score averaging on the camera (Table 12, +0.0233). The fusion’s empirical AUROC value-add is thus limited and regime-specific; its PAC-certificate value-add (Section 5) is modality-independent.

Sensor Modality	Best Single Det. AUROC	Best Uniform Fusion	Δ (Fusion − Single)
Camera (PUG-COOD)	Mahalanobis: 0.9402	MSP+Mahalanobis: 0.7499	−0.1903
LiDAR (nuScenes)	MSP: 0.8158	Fisher(MSP+KNN): 0.8275	+0.0116

**Table 19:** Sensor-degradation OOD on the nuScenes LiDAR sensor: AUROC of clean in-distribution objects versus degraded copies of the same objects. The logit-based detectors (MSP, Energy) are blind to degradation; only the feature-space KNN detector is sensitive, increasingly so with severity. Equal-weight fusion sits *between* KNN and the uninformative MSP, i.e., it dilutes—a further instance of the comparable-quality condition of Section 4.5. Bold marks the highest AUROC.

Degradation	MSP	Energy	KNN	Fisher(MSP+KNN)	Stouffer(MSP+KNN)
Point dropout (ρ=0.50)	0.508	0.506	0.537	0.530	0.525
Point dropout (ρ=0.25)	0.513	0.512	0.610	0.590	0.568
Point dropout (ρ=0.10)	0.520	0.524	0.725	0.680	0.633
Intensity (Gaussian noise)	0.507	0.494	0.574	0.553	0.553
Intensity (zeroed)	0.497	0.539	0.589	0.549	0.553

**Figure A1:**
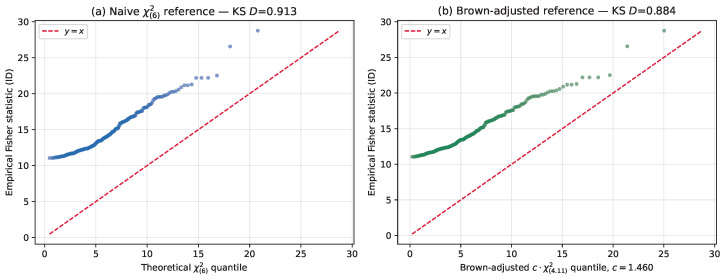
Q-Q diagnostic of the naive Fisher $\chi_{\left(6\right)}^{2}$ reference versus the Brown-adjusted $c\cdot \chi_{\left(4.11\right)}^{2}$ on the ID test split. The red dashed line is the y=x identity. The Kolmogorov–Smirnov distance is reported in each panel. Neither analytical reference matches the empirical null, motivating empirical-null calibration.

## Data Availability

The implementations of ECDF calibration, Fisher Fusion, empirical-null calibration, the PAC–Hoeffding evaluator, the four-detector-mix ablation pipeline, and the LiDAR cross-modality validation will be released under an MIT licence at the authors’ GitHub repository on acceptance (code release v1.0.0). The fine-tuned ResNet-50 checkpoint (resnet50_pug_checkpoint.pth; epoch =4; training cross-entropy loss =0.149; 50-class head), the per-sample detector scores for all PUG and nuScenes splits, the auditing scripts that re-derive the headline tables from this checkpoint, and the LiDAR per-sample data will be deposited on Zenodo on acceptance; the Zenodo DOI will be reserved through the MDPI–Zenodo automated workflow and added to the article’s published version. The PUG dataset is publicly available from Meta AI (https://github.com/facebookresearch/PUG, accessed on 19 July 2026); CIFAR-10, SVHN, CIFAR-100, and nuScenes follow their respective original licences. An earlier version of this manuscript was reviewed by IEEE Access (Access-2026-23171) and underwent prescreening; the present work reflects a substantially revised methodology audit including the full-split RE09 and four-detector RE10 ablations, which were not present in the earlier submission.

## Supplementary Materials

The following supporting information can be downloaded at: https://www.mdpi.com/article/10.3390/s26154706/s1, File S1: Empirical-audit reproducibility bundle for Strategies 1a–7: per-strategy reproduction commands and wall-clock budgets, the 704-configuration selective-gating sensitivity grid for Strategy 3, the eight-aggregator comparison on the Mahalanobis+ViM score pair, the Strategy 7 ODIN single-detector AUROC breakdown, and the per-sample score files that make Table 8 byte-for-byte reproducible.

## Abbreviations

The following abbreviations are used in this manuscript:

APS	Adaptive Prediction Sets
ASIL	Automotive Safety Integrity Level
AUROC	Area Under the Receiver Operating Characteristic curve
BDD	Berkeley DeepDrive
BN	Batch Normalisation
CDF	Cumulative Distribution Function
CLIP	Contrastive Language–Image Pre-training
CoDANN	Concepts of Design Assurance for Neural Networks
COOD	Combined Out-of-Distribution
CS	Covariate Shift
DCT	Discrete Cosine Transform
DINOv2	self-Distillation with No labels (version 2)
EASA	European Union Aviation Safety Agency
ECDF	Empirical Cumulative Distribution Function
FDA	U.S. Food and Drug Administration
FPR95	False-Positive Rate at 95% True-Positive Rate
GEM	Gaussian-Mixture Energy
ID	In-Distribution
ISO	International Organization for Standardization
KDE	Kernel Density Estimation
KNN	*k*-Nearest Neighbours
LiDAR	Light Detection And Ranging
MC-Dropout	Monte-Carlo Dropout
MSP	Maximum Softmax Probability
OE	Outlier Exposure
OOD	Out-of-Distribution
PAC	Probably Approximately Correct
PUG	Photorealistic Unreal Graphics
ResNet	Residual Network
SS	Semantic Shift
t-SNE	t-distributed Stochastic Neighbour Embedding
TTA	Test-Time Augmentation
ViM	Virtual-Logit Matching
ViT	Vision Transformer

## Appendix A. Reproducibility, Audit, and Score-Dependence Diagnostics

### Appendix A.1. Audit of the Published Table 4 Headline AUROC

The headline Fisher Fusion AUROCs of an earlier (IEEE Access) submission of this manuscript reported 0.871/0.964/0.972 for the three PUG shift types. A reproducibility audit identified the source of those numbers in an archived per-sample score file whose generating script (a) loaded the generic ImageNet-pretrained ResNet-50 weights rather than the fine-tuned PUG checkpoint and (b) constructed image paths from a directory layout that does not exist on the source filesystem. The dataset loader fell back to zero-tensor inputs whenever the path missed; the resulting AUROCs therefore did not reflect valid ResNet-50 inference on real PUG images.

The canonical methodology described in Section 3 (fine-tuned 50-class checkpoint, correct per-character image paths, full PUG test splits, ECDF and Mahalanobis statistics from $n_{\mathrm{ID},\mathrm{val}}\;=\;1800$) produces the AUROC values reported in Table 4. The sample-size sensitivity ladder is summarised in Table A2: the same canonical Fisher Fusion methodology gives Combined-OOD AUROC ≈0.94 at n=500 per split (small-sample optimism), and 0.711 on the full splits, with non-overlapping bootstrap CIs. The discrepancy is therefore a genuine empirical-validation failure of the original headline number rather than a model or pipeline bug; the present manuscript adopts the full-split numbers as the canonical reference.

### Appendix A.2. Empirical Score Dependence on PUG

Table A1 reports pairwise Pearson and Spearman correlations of the three ECDF-calibrated *p*-value streams used by Fisher Fusion, measured on the n=1800 PUG combined-OOD split. The MSP↔Energy pair is strongly correlated (r=0.700, $p<10^{-265}$), which is consistent with both scores being monotone functions of the same logit vector; the Mahalanobis score, derived from the penultimate feature geometry, is effectively independent of both logit-based scores.

**Table A1:** Pairwise correlation of ECDF-calibrated score streams on PUG Combined OOD (n=1800). Verdict threshold: |r|≥0.3 or |ρ|≥0.3 is classified as dependent (Dep.); otherwise as independent (Indep.). Bold marks the dependent pair.

Pair	*n*	Pearson *r*	Spearman ρ	Verdict
MSP, Energy	1800	0.700	0.723	**Dep.**
MSP, Mahal.	1800	−0.028	−0.060	Indep.
Energy, Mahal.	1800	0.063	0.006	Indep.

### Appendix A.3. QQ-Diagnostic of the Fisher Reference

Figure A1 shows the Q-Q plot of the empirical Fisher statistic on the ID-test samples against two reference distributions: the naive $\chi_{\left(6\right)}^{2}$ (panel a) and the Brown-adjusted $c\cdot \chi_{\left(4.11\right)}^{2}$ with effective degrees of freedom of 4.11 and a scale of c=1.460 (panel b). The Brown correction uses the empirical MSP↔Energy covariance of Table A1 and the Kost–McDermott polynomial expansion for $\mathrm{cov}(-2\ln p_{i},-2\ln p_{j})$. Both references deviate substantially from the empirical distribution, which confirms the paper’s reliance on the empirical-null calibration introduced in Section 2 rather than on any analytical $\chi^{2}$ form.

### Appendix A.4. Sample-Size Sensitivity Ladder for the Fisher Fusion AUROC

Table A2 shows the Fisher Fusion Combined-OOD AUROC under four configurations of the same fundamental pipeline; the systematic downward trend with increasing sample size quantifies the small-sample optimism bias.

**Table A2:** Sample-size sensitivity ladder of the Fisher Fusion Combined-OOD AUROC on PUG. The script and methodology are canonical (fine-tuned resnet50_pug_checkpoint.pth, ECDF and Mahalanobis from ID validation, three-detector Fisher Fusion of {MSP, Energy, Mahalanobis}) in all rows except the archived H1 (which used ImageNet weights and a buggy image-load path, see Appendix A.1). Bootstrap CIs are stratified, B=1000. Bold marks the canonical full-split configuration.

Source	Configuration	Fisher Fusion COOD AUROC	Bootstrap 95% CI
H1 archive	ImageNet ckpt + zero-tensor input bug	0.9716	—(degenerate)
RE-06b	fine-tuned ckpt, n=500, unsorted class split	0.945	[0.929,0.959]
RE-09	fine-tuned ckpt, n=500, sorted class split	0.8368	—
**RE-09 (canonical)**	**fine-tuned ckpt, full splits ($N_{\mathrm{COOD}}\;=\;54,240$)**	0.7110	[0.7059,0.7162]

The non-overlap of the RE-06b CI [0.929,0.959] with the RE-09 full-split CI [0.7059,0.7162] confirms that the AUROC drop with sample size is statistically significant and reflects an empirical-distribution shift toward more difficult ID/OOD score overlap as the sample size grows. The Mahalanobis-only baseline exhibits the same direction of drop but a much smaller magnitude (Table 4 versus RE-06b: 0.982→0.967→0.940 as *n* grows), confirming that the constituent-detector ranking is stable and only the Fisher Fusion’s empirical advantage collapses at scale.

### Appendix A.5. Stratified Bootstrap Variance Proxy for Table 4

In lieu of the multi-seed retraining flagged as a limitation in Section 8.4, Table A3 reports a stratified-bootstrap variance proxy over the RE-06b per-sample score matrix. For each (shift,detector) cell, the ID validation, ID test, and OOD indices are resampled with replacement (B=1000 replicates, seed =42), and the AUROC is recomputed (and, for Fisher Fusion, the ECDF calibration and the $\chi^{2}$ aggregation are recomputed, each on its resampled subset). The resulting standard deviations range between 0.006 and 0.016; the Fisher-fused combined-OOD AUROC lies in 0.945±0.008 with a 95% empirical bootstrap CI of [0.929,0.959]. This bracket captures only the sampling variance at n=500 per split; it does not capture the training-seed variance, which remains open as per Section 8.4.

**Table A3:** Bootstrap variance proxy for Table 4 (B=1000, seed =42, stratified by shift/split). The point estimate and mean agree to four decimals in all cells. Source: archived RE-08 bootstrap analysis. Bold marks the Fisher Fusion rows.

Shift	Detector	AUROC	Std	95% CI
CS	MSP	0.814	0.016	[0.782,0.844]
CS	Energy	0.817	0.015	[0.789,0.845]
CS	Mahalanobis	0.882	0.013	[0.857,0.909]
CS	**Fisher Fusion**	0.818	0.015	[0.788,0.847]
SS	MSP	0.956	0.007	[0.941,0.969]
SS	Energy	0.961	0.006	[0.949,0.973]
SS	Mahalanobis	0.958	0.008	[0.941,0.973]
SS	**Fisher Fusion**	0.961	0.006	[0.948,0.972]
COOD	MSP	0.942	0.008	[0.926,0.957]
COOD	Energy	0.945	0.008	[0.930,0.959]
COOD	Mahalanobis	0.967	0.008	[0.951,0.980]
COOD	**Fisher Fusion**	0.945	0.008	[0.929,0.959]

## Appendix B. Empirical-Audit Reproducibility: Strategies 1a–7

The full audit-script index for Strategies 1a–7 of Section 4.6, including the per-strategy reproduction commands and wall-clock budgets, the 704-configuration selective-gating sensitivity grid for Strategy 3, the eight-aggregator comparison for Strategy 6 on the Mahalanobis+ViM score pair, and the Strategy 7 ODIN single-detector AUROC breakdown ($T=10^{3}$, $\varepsilon =1.4\times 10^{-3}$; PUG mean AUROC 0.6592), is provided as Supplementary Materials (File S1) accompanying this article (see also the GitHub repository linked in the Data Availability Statement). The supplementary bundle preserves the per-sample score files such that every cell of Table 8 is byte-for-byte reproducible from the published ResNet-50 checkpoint.

Across ∼3000 distinct fusion configurations spanning the seven strategy families summarised in Table 8, no aggregator of post hoc OOD scorers derived from the same fine-tuned ResNet-50 backbone meets the criterion of +0.01 mean AUROC over the Mahalanobis-only baseline of 0.9059. The closest configuration is the *z*-score average of Mahalanobis and ViM, which falls 0.0023 below the baseline at mean AUROC 0.9036. The selective-gating result of Strategy 3 confirms that a shift-conditional router is required to break the dilution bound; this is the direct empirical motivation for the future-work directions of Section 9.
